# The emergence and successful elimination of SARS-CoV-2 dominant strains with increasing epidemic potential in Taiwan’s 2021 outbreak

**DOI:** 10.1016/j.heliyon.2023.e22436

**Published:** 2023-11-20

**Authors:** Chin-Rur Yang, Sui-Yuan Chang, Yu-Nong Gong, Chung-Guei Huang, Tsung-Hua Tung, Wei Liu, Ta-Chien Chan, Kuo-Sheng Hung, Hung-Sheng Shang, Jih-Jin Tsai, Chuan-Liang Kao, Hui-Lin Wu, Li-Yu Daisy Liu, Wan-Yu Lin, Yi-Chin Fan, Chwan-Chuen King, Chia-Chi Ku

**Affiliations:** a Graduate Institute of Immunology, College of Medicine, National Taiwan University, 1 Jen-Ai Road Section 1, Taipei, 10051, Taiwan, ROC; b Department (Dept.) of Clinical Laboratory Sciences and Medical Biotechnology, College of Medicine, National Taiwan University, Taipei, 10051, Taiwan, ROC; c Dept. of Laboratory Medicine, National Taiwan University Hospital, Taipei, 10051, Taiwan, ROC; d Research Center for Emerging Viral Infections, College of Medicine, Chang Gung University, Taoyuan, 33302, Taiwan, ROC; e Dept. of Laboratory Medicine, Linkou Chang Gung Memorial Hospital, Taoyuan, 33302, Taiwan, ROC; f Dept. of Medical Biotechnology and Laboratory Science, College of Medicine, Chang Gung University, Taoyuan, 33302, Taiwan, ROC; g Institute of Epidemiology and Preventive Medicine, College of Public Health, National Taiwan University, NTU 17 Xu-Zhou Road, Taipei, 10055, Taiwan, ROC; h Dept. of Health, Taipei City Government, Taipei, Taiwan, ROC; i Research Center for Humanities and Social Sciences, Academia Sinica, Taipei, 11529, Taiwan, ROC; j Center for Precision Medicine and Genomics, Tri-Service General Hospital, National Defense Medical Center, Taipei, 11490, Taiwan, ROC; k Division of Clinical Pathology, Dept. of Pathology, Tri-Service General Hospital, National Defense Medical Center, Taipei, 11490, Taiwan, ROC; l School of Medicine, College of Medicine, Kaohsiung Medical University, Kaohsiung, 80708, Taiwan, ROC; m Tropical Medicine Center, Kaohsiung Medical University Hospital, Kaohsiung, 80756, Taiwan, ROC; n Division of Infectious Diseases, Department of Internal Medicine, Kaohsiung Medical University Hospital, Kaohsiung, 80756, Taiwan, ROC; o Hepatitis Research Center, National Taiwan University Hospital, Taipei, 10051, Taiwan, ROC; p Graduate Institute of Clinical Medicine, College of Medicine, National Taiwan University, Taipei, 10051, Taiwan, ROC; q Division of Biometry, Department of Agronomy, National Taiwan University, Taipei, 10617, Taiwan, ROC

**Keywords:** SARS-CoV-2, Viral variants, Whole-genome sequencing, Community outbreak, Reproduction number, Transmission, Bioinformatics, Spatio-temporal analysis, Epicenter, Taiwan

## Abstract

Taiwan’s experience with severe acute respiratory syndrome coronavirus (SARS-CoV) in 2003 guided its development of strategies to defend against SARS-CoV-2 in 2020, which enabled the successful control of Coronavirus disease 2019 (COVID-19) cases from 2020 through March 2021. However, in late-April 2021, the imported Alpha variant began to cause COVID-19 outbreaks at an exceptional rate in Taiwan. In this study, we aimed to determine what epidemiological conditions enabled the SARS-CoV-2 Alpha variant strains to become dominant and decline later during a surge in the outbreak. In conjunction with contact-tracing investigations, we used our bioinformatics software, CoVConvert and IniCoV, to analyze whole-genome sequences of 101 Taiwan Alpha strains. Univariate and multivariable regression analyses revealed the epidemiological factors associated with viral dominance. Univariate analysis showed the dominant Alpha strains were preferentially selected in the surge’s epicenter (p = 0.0024) through intensive human-to-human contact and maintained their dominance for 1.5 months until the Zero-COVID Policy was implemented. Multivariable regression found that the epidemic periods (p = 0.007) and epicenter (p = 0.001) were two significant factors associated with the dominant virus strains spread in the community. These dominant virus strains emerged at the outbreak’s epicenter with frequent human-to-human contact and low vaccination coverage. The Level 3 Restrictions and Zero-COVID policy successfully controlled the outbreak in the community without city lockdowns. Our integrated method can identify the epidemiological conditions for emerging dominant virus with increasing epidemiological potential and support decision makers in rapidly containing outbreaks using public health measures that target fast-spreading virus strains.

## Highlights

• Frequent human-to-human contact at the epicenter selected dominant SARS-CoV-2 strains.
• Dominant SARS-CoV-2 strains had the highest Rt values under specific epidemiological conditions.
• Level 3 Restrictions and Zero-COVID Policy were effective without lockdown.
• Individual- and population-based prevention measures contained fast-spreading dominant virus strains.
• Preventing cluster cases that would have aided viral selection effectively defended against VOCs.

## Graphical abstract

This figure summarizes our major findings in this study. Before the outbreak (T0, Pre-outbreak), the imported Alpha variant strains were heterogeneous with high viral genome divergence. However, such diversity significantly decreased during the T1 period (versus T0, p < 0.0001), when the dominant virus strains with selective advantages appeared. We also investigated the epidemiological conditions in Taiwan that facilitated the emergence of the predominant virus strain in the T1 period. The effective reproductive numbers over time (Rt) for the viruses from the imported cases were all zero at T0 period before the outbreak. However, the mean Rt values of the viruses from the pilots to quarantine hotel staff and subsequent dominant virus strains in the community (i.e. same sequence identities as the ID-3445/1263/1186) increased rapidly. Specific epidemiological conditions, including unmasked dining in many teahouses, and customers’ movement across teahouses, helped the dominant Alpha variant strains with a selective advantage. This study demonstrated that natural selection of a dominant virus strain (prior to immune selection) progressed in three stages: (1) selection started from a diverse virus pool (i.e. imported viruses at T0), (2) selection advantages increased through virus replication, in which the progeny virus had more advantages than its parent virus, and (3) selection of a fast-spreading virus strain through human-to-human transmission when a community had suitable epidemiological conditions (i.e. our T1 period and epicenter). Most importantly, COVID-19 cases dropped sharply alongside the two important population-intervention strategies (Level 3 Restrictions and Zero-COVID policy).

**Figure:**
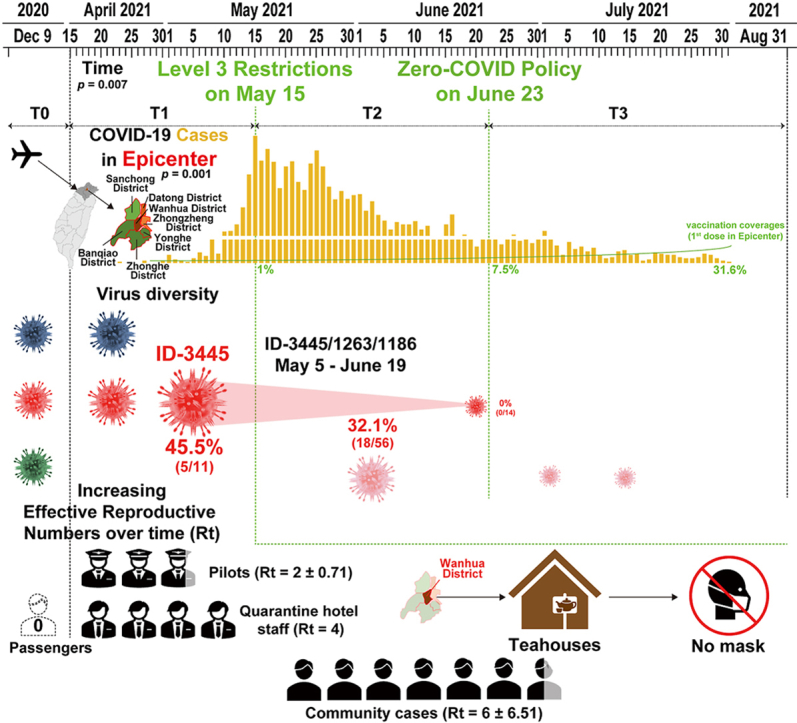

## 1 Introduction

The Taiwan Centers for Disease Control (Taiwan CDC) had learned hard lessons during the SARS-CoV outbreaks in 2003 [1]. Thus, when SARS-CoV-2, a wide-spreading virus, began wreaking global havoc, Taiwan quickly responded to the pandemic with early border control measures on December 31, 2019 [2]. As a result, although SARS-CoV-2 caused over 220 million COVID-19 cases and nearly 4.58 million deaths worldwide through September 4, 2021 [3], Taiwan did not experience any large COVID-19 outbreaks. Rather, only three incidences of limited community spread occurred in Taiwan from 2019 to mid-April 2021.

Continuous mutations in the SARS-CoV-2 viral genomes have evolved different lineages with higher transmissibility and increased virus fitness in the human host. Among the variants of concern (VOCs), the Alpha variant (B.1.1.7 lineage) with the highest relative fitness [4] has exacerbated pandemic concerns ever since its initial detection in the UK in September 2020 [5]. In December 2020, the Alpha variant was imported into Taiwan for the first time. After a few controllable waves, the re-introduction of the Alpha variants began to cause new COVID-19 cases at an unprecedented rate in late April, driving 14,311 total indigenous cases. However, within 100 days of implementing the Level 3 Restrictions (started on May 15, 2021) and the Zero-COVID Policy (started on June 22, 2021) to control this outbreak, Taiwan reached zero indigenous cases and finally ended this outbreak on September 4, 2021.

Contact-tracing investigations confirmed several cluster cases before the surge in 2021. Three key questions thus arose: Were the different strains of SARS-CoV-2 Alpha variants from various early-outbreak transmission chains associated with igniting the community outbreak? What epidemiological factors facilitated the fast spread or hindrance of Alpha variant strains in the community? What lessons have we learned from how Taiwan controlled this outbreak that could help other countries quickly contain fast-spreading virus strains?

By employing our in-house developed software (CoVConvert and IniCoV), which integrated epidemiological information and whole-genome sequences of Taiwan SARS-CoV-2, this study aimed to (1) investigate the outbreak’s spatiotemporal trends; (2) characterize the mutational dynamics of SARS-CoV-2 strains isolated from each at the early-outbreak transmission chains before and during wide community-spread across different outbreak periods, and (3) identify epidemiological factors associated with the upsurge and decline of community-spread dominant Alpha strains. We demonstrated that among multiple introductions of SARS-CoV-2 Alpha strains to Taiwan during the early-outbreak period, one dominant virus strain was highly associated with the subsequent occurrence of the large outbreak. The dominance of such a particular strain declined after the introduction of population-based intervention strategies (e.g., Level 3 Restrictions and the Zero-COVID Policy) and reinforcement of individual-based preventive measures (e.g., wearing facemasks, keeping social distance, etc.) [2]. Timely containment of viral spread through public health efforts is the key to curbing new transmission waves of virus strains with high transmissibility. We hope this integrated analysis platform enables efficient monitoring of viral dynamic changes through genomic surveillance [6,7].

Our findings on necessary epidemiological conditions that facilitate the emergence of novel viral strains with increasing epidemic potential and epidemiological risk assessment can prevent future pandemics. The significance of this study lies in the development of a novel integrated analysis platform to comprehend the dynamic changes in viral whole genome under various control measures and identified the factors that are associated with the emergence, rise, and decline of dominant virus strains. Therefore, with full understanding of these factors, decision-makers can quickly minimize the health threat of the dominant virus strains.

## 2 Methods

### 2.1 Study design

We analyzed 16,132 laboratory-confirmed SARS-CoV-2-positive cases from January 11, 2020 to September 4, 2021 in Taiwan, then focused on 14,636 cases (14,311 indigenous cases) from the 2021 outbreak (April 16–September 4). As the majority of outbreak cases (86.27%, 12,346/14,311) occurred in Taipei, New Taipei, and Taoyuan cities, the spatiotemporal distributions of cases in these cities across four different time periods were plotted using Microsoft Power BI.

**Fig. 1:**
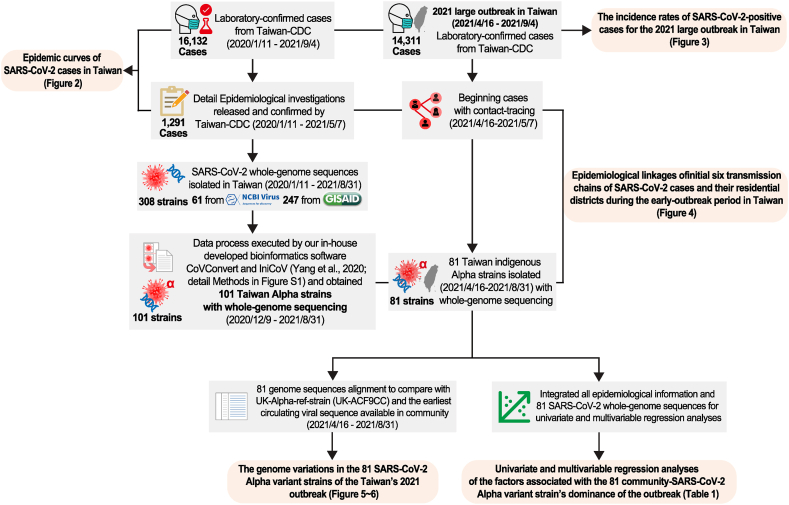
Flow diagram of study design to analyze SARS-CoV-2-positive cases in Taiwan from January 11, 2020 to September 4, 2021. We used CoVConvert to check data quality and obtained different reading frames for IniCoV to identify polygenetic consensus signatures. Detailed steps are described in Fig. S1.

To search for possible viral sequence differences that launched this outbreak, we combined whole-genome sequences of 101 Taiwan SARS-CoV-2 Alpha variants from COVID-19 positive patients with their onset dates from December 9, 2020 to August 31, 2021 (Table S4). These included 12 imported strains from before the outbreak (T0, pre-outbreak) and an additional 12 strains (9 imported and 3 indigenous strains) from the beginning of this outbreak (T1a, April 16, 2021–May 7, 2021; early-outbreak). Each confirmed case included comprehensive contact-tracing through joint efforts of epidemiologists from local Departments of Health (DOH) and Taiwan CDC. The integrated information helped investigate the early transmission chains that might be associated with subsequent community spread (81 indigenous strains from patients’ onset of illness occurred between May 7, 2021 and August 31, 2021). These 81 strains involved three time periods based on public health interventions: T1 (April 16–May 14; pre-Level 3 Restrictions), T2 (May 15–June 22; post-Level 3 Restrictions, but pre-Zero-COVID Policy), and T3 (June 23–August 31; post-Zero-COVID Policy) were analyzed to look for whether a dominant virus strain was persistently spreading in the community. Finally, we applied univariate and multivariable analyses to search for epidemiological factors attributed to the appearance of the dominant virus strains (Fig. 1).

### 2.2 Prevention and control measures

The government has rigorously enforced the Level 3 Restrictions and the Enhanced Zero-COVID Policy. Level 3 Restrictions started on May 15, 2021 for Taipei City and New Taipei City (the two main outbreak-affected cities) and extended nationwide on May 19, 2021, and finally ended on February 28, 2022. This policy included closing leisure and entertainment venues; avoiding unnecessary movement, activities, and gatherings; and stopping of all family or social gatherings involving five or more people indoors or ten or more people outdoors. All public and commercial areas must be closed, except for medical care, public services, and living maintenance [8,9]. As the daily COVID-19 case numbers were noticeably growing and surrounding the largest wholesale poultry market in Taipei City, the city mayor initiated “the Zero-COVID Policy” on June 22, 2021 to detect more asymptomatic and pre-symptomatic infections via community-based mass PCR screening [[8], [9], [10]]. The Zero-COVID Policy included two prevention measures: (1) all laboratory-confirmed SARS-CoV-2-positive patients had to go to the hospital for treatment if they had COVID-19 symptoms, or were required to stay at quarantine hotels if they were asymptomatic or pre-symptomatic, to prevent intrafamilial transmission, and (2) enhanced epidemiological investigation and contact tracing was performed on all SARS-CoV-2-positive patients, plus further PCR testing on all their possible contacts or people who had visited the same sites, to achieve thorough and fast containment of all possible high-risk persons who had contacts with SARS-CoV-2-positive patients.

### 2.3 Study populations of SARS-CoV-2-positive cases in Taiwan

**Fig. 2:**
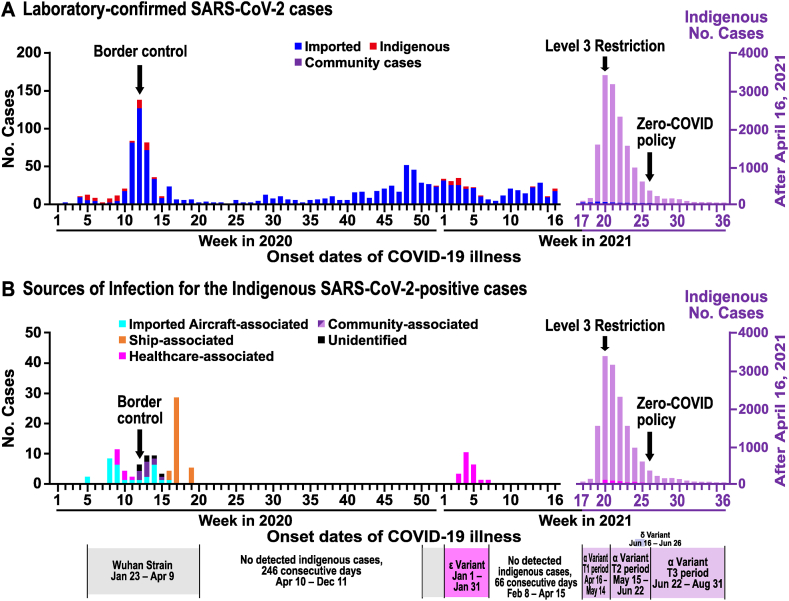
Epidemic curves of laboratory-confirmed SARS-CoV-2 cases plotted with three major government countermeasures in Taiwan from January 1, 2020, to September 4, 2021. The weekly numbers of laboratory-confirmed SARS-CoV-2 cases from the 1st week of 2020 to the 36th week of 2021 (i.e. 4 September 2021 when the daily case number dropped below 10) were obtained from Taiwan CDC Open Data Portal (https://data.cdc.gov.tw/en). The bar graphs show the distribution of cases based on the onset weeks, and the arrows indicate when countermeasures were implemented (Detail described in Methods). The confirmed indigenous cases caused by the three variants of SARS-CoV-2 are: (1) Alpha variants (14,311 cases, April 16-September 4, 2021), (2) Epsilon variants (19 cases, January 1-January 31, 2021), and (3) Delta variants (15 cases, June 16-June 26, 2021). (A) Weekly numbers of confirmed imported (shiny blue bars), indigenous (red bars), and the 2021 large outbreak (light purple bars, from the 16th to 36th week of 2021). The two waves of the imported cases involved western holidays: 1) the 48th week of 2020 (early December, 50 cases after Thanksgiving holidays) through the 1st week of 2021 (38 cases after New Year’s holidays), and 2) 16th-19th week of 2021 after Spring breaks (mid-April, mean ± S.D.: 29.5 ± 12.95 cases/week). (B) Sources of the infection for indigenous cases involved into five major risk groups from January 1, 2020, to September 4, 2021. Before the 2021 large outbreak (1st week of 2020 to the 7th week of 2021): 1) Imported Aircraft-associated cases (light blue bars, contact history with imported cases) = 29/114, 25.4%, 2) Healthcare-associated cases (magenta bars) = 30/114, 26.3% (9 cases in the 9th −11th weeks of 2020 and 21 cases in the 2nd -6th weeks of 2021), 3) Community-associated cases (purple bars, indigenous cases who had no travel history three days before the onset of illness) = 12/114, 10.5%, 4) Ship-associated cases (orange bars, cruise ships and naval crews) = 36/114, 31.6% (36 cases in the 16th −19th weeks of 2020), and 5) Cases with unidentified sources (black bars, no clear sources of infection following thorough epidemiological investigation) = 7/114, 6.2%. During the 2021 large outbreak (17th week of 2021 to the 36th week of 2021): 1) Healthcare-associated cases (magenta bars) = 244/14,311, 1.7% (244 cases in the 20th −25th weeks of 2021), 2) Community-associated cases and cases with unidentified sources (light purple bars, indigenous cases who had no travel history three days before the onset of illness) = 14,067/14,311, 98.3%.

All the laboratory-confirmed SARS-CoV-2-positive cases in 2021 were tested using real-time RT-PCR on patients exhibiting or being suspected of COVID-19 clinical symptoms. We plotted an overall epidemic curve of total imported and indigenous SARS-CoV-2-positive cases from January 1, 2020 to September 4, 2021 (Fig. 2A). According to information released from local DOH and confirmed by Taiwan CDC, we categorized infection sources for indigenous cases into five major risk groups [2] (imported-aircraft-associated, healthcare-associated, community-associated, ship-associated, and unidentified sources) (Fig. 2B).

### 2.4 SARS-CoV-2 genome sequence alignment and mutation analyses

The 308 whole-genome sequences of SARS-CoV-2 in Taiwan were retrieved from NCBI-Virus and GISAID-EpiCoV databases. These sequences were obtained from patients who had onset dates of COVID-19 illness between January 11, 2020 and August 31, 2021. We used our in-house developed analytical tools, CoVConvert and IniCoV, to process and analyze these SARS-CoV-2 sequences [11] (Fig. S1, S2). CoVConvert rearranged the sequences of the 101 Taiwan Alpha variants to ensure data quality, then aligned and translated them into three polypeptides from three reading frames. Next, IniCoV automatically divided the translated polypeptides into 31 proteins (Fig. S3) for each viral strain, combined them with individual epidemiological information, and subsequently compared these 101 strains with the Alpha variants' reference strain (UK-MILK-ACF9CC, referred to as “UK-Alpha-ref-strain”) to analyze any residue differences among these strains involving three epidemiological groups: (1) the 12 imported strains before the outbreak (T0), (2) the initial 12 strains from early-outbreak (T1a), and (3) the remaining 77 strains (T1b, T2, T3).

### 2.5 Contact-tracing investigations and transmissibility analysis of the early-outbreak cases in the Taiwan’s 2021 outbreak

To measure viral transmissibility, we applied epidemiological contact-tracing investigations to compare the effective reproductive numbers over time (Rt) of those early Alpha variant cases (Fig. 4 and Table S1). The range and mean ± standard deviation (SD) values of Rt were calculated for three groups: cases before the outbreak, airport-associated cases (pilots, hotel staff) in the early-outbreak, and community-associated cases. Significant differences among the three groups were tested using one-way ANOVA.

### 2.6 Univariate and multivariable regression analyses of factors associated with SARS-CoV-2 strains' dominance in the outbreak

To understand the significant factors were associated with the dominant SARS-CoV-2 strains, we used four R packages (Supplementary Methods) to examine the 81 Taiwan indigenous strains for univariate analysis. The nine factors included: (1) epidemic periods, (2) epicenter, (3) vaccination coverage, (4) public transport ridership, (5) numbers of daily cases, (6) population size, (7) population density, (8) age, and (9) gender. Factors 3–8 were separated into “high” and “low” groups based on the median. We used Fisher’s exact test to assess all factors between subgroups and obtained the crude odds ratios (cOR) with 95% confidence intervals (CIs). All statistically significant factors (p < 0.05) were checked with correlations by calculating variance inflation factors (VIF) before running the multivariable regression (Table S6). The best-fitting model was selected from the candidate models generated from the stepwise (backward and forward) search method by choosing the lowest Akaike information criterion (AIC) value (Table S7). We also reported the adjusted ORs (aOR) with 95% CIs and *p*-values from the best-fitting model to present the factors associated with the dominant indigenous strains of Alpha variants.

## 3 Results

### 3.1 Characteristics in SARS-CoV-2-positive cases before and after Taiwan’s 2021 outbreak

In Taiwan, imported SARS-CoV-2 cases caused by Wuhan strains increased in the 4th-6th weeks of 2020 (3–8 cases per week), first peaked in the 12th week (mid-March, 125 cases) when Taiwanese students returned from Europe and the USA, and then quickly declined after the government implemented strict border controls on March 19, 2020 (Fig. 2A). Later, imported cases of Alpha variants rose slightly again in two waves from the 48th week of 2020 through the 1st week of 2021 (post-holiday season), and the 16th–19th week (around spring breaks) of 2021.

For indigenous cases, two small waves occurred in the 4th–15th weeks of 2020 and the 51st week of 2020 to the 7th week of 2021, which were caused mainly by the Wuhan strains (114 cases) and Epsilon variants (19 cases), respectively. No indigenous cases occurred from the 8th-16th weeks of 2021. Up until April 16, 2021 (the 17th week), the beginning cases, including pilots and hotel staff were all airport-associated cases, after which the large outbreak occurred (peaking at 3363 cases in mid-May 2021, the 20th week) (Fig. 2A).

Sources of the infection for the 114 prior-indigenous cases (January 22, 2020 to April 15, 2021) before 2021's outbreak involved different risk groups, and community-associated plus unidentified cases accounted for 16.7% (19/114) of the total. In contrast, most community-associated plus unidentified cases during the outbreak (April 16, 2021–September 4, 2021) significantly increased and accounted for 98.3% (14,067/14,311) of the total (p < 0.0001) (Fig. 2B).

### 3.2 Characterization of Taiwan’s 2021 outbreak

**Fig. 3:**
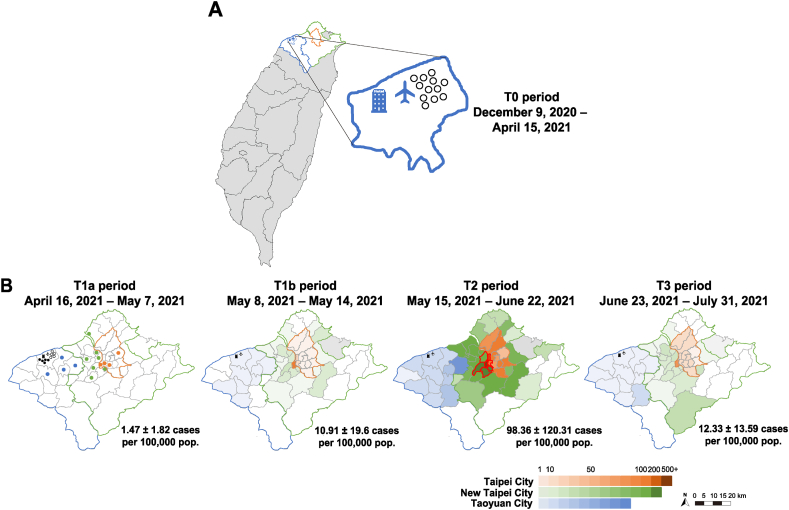
The incidence rates of laboratory-confirmed SARS-CoV-2 cases in the three major affected cities and other areas of Taiwan from Pre-outbreak and during the large 2021 outbreak (from December 9, 2020 through July 31, 2021). (A) Pre-outbreak (T0, December 9, 2020 through April 15, 2021).Symbols and lines shown the Taoyuan International Airport and quarantine hotel in Dayuan District, and imported cases (in the circle). (B) During the outbreak (T1-T3, April 16, 2021 through July 31, 2021). The colour gradients show the incidence rate (per 100,000 residents) in each district in the three major affected cities across five different time periods [T1a (April 16, 2021–May 7, 2021; early-outbreak), T1b (May 8, 2021–May 14, 2021; pre-Level 3 Restrictions), T2 (May 15, 2021–June 22, 2021; post-Level 3 Restrictions, but pre-Zero-COVID Policy), T3 (June 23, 2021–July 31, 2021; post-Zero-COVID Policy]. The early-outbreak (T1a) cutting time point on May 7 because the last pilot case ID-1183 and ID-1187 who had onset dates on May 6. The Daily mean numbers, and red lines show the six districts neighboring the area where the epidemic began (Wanhua District). The “epicenter” of this outbreak was defined as the district with the highest incidence and its bordering districts (Table S2). Data on district-specific population sizes was obtained using Taiwan household registry information from the Ministry of Interior population sizes in May 2021 were 2,574,704 in Taipei City, 4,026,019 in New Taipei City, and 2,270,939 in Taoyuan City (https://www.ris.gov.tw/app/en/346). The numerator represents number of new cases occurring at that specific time period in the same studied district as the denominator. The monthly incidence rates are shown as “mean ± SD” before and after the 2021 outbreak: Taipei City: 0.152 ± 0.161 vs 46.691 ± 56.311 (p < 0.0001), New Taipei City: 0.162 ± 0.066 vs 42.161 ± 49.067 (p < 0.0001), and Taoyuan City: 0.265 ± 0.303 vs 8.288 ± 7.606 (p < 0.0001).

There were 12 imported cases, but no indigenous ones reported in the T0 period (Fig. 3A). However, starting on April 16, the emergence of sporadic episodes of SARS-CoV-2-positive clusters associated with the airport and the quarantine hotel (Fig. 3B, T1a) led to a large outbreak. Mean weekly numbers and the monthly incidence rate of SARS-CoV-2-positive cases rapidly increased in Taipei, New Taipei, and Taoyuan cities. The mean incidence rate (per 100,000 population) was 10.91 ± 19.6 in T1b and peaked at 98.6 ± 120.31 in T2. The highest case incidence was observed in Taipei (with the largest population) between May 7–May 14. Total daily cases in these three cities peaked at 495 cases on May 15, when Taipei and New Taipei cities implemented the Level 3 Restrictions. Shortly thereafter, in early June, daily case numbers in both cities dropped below 100. On June 23, Taipei City launched the Zero-COVID Policy, and daily case numbers had decreased again to ten cases as of July 10 (Fig. S3). The mean incidence rate declined to 12.33 ± 13.19 (Fig. 3B, T3). It took 100 days from the peak on May 15 to reach zero indigenous cases on August 22 in all cities and the epidemic finally ended on September 4, without lockdowns.

Based on the spatiotemporal analysis of diffusion patterns over time, the Wanhua District in Taipei City had the highest incidence rates across all periods. Its six neighboring districts, which had the second and third highest incidence rates from April 16 to June 22, were considered the epicenter (Table S2). Subsequently, cases spread rapidly from the epicenter to other districts with larger population sizes and greater population densities (Fig. 3B, T2 and T3).

### 3.3 Integrating whole-genome sequence analysis and contact-tracing investigations to search for dynamic sequence changes

**Fig. 4:**
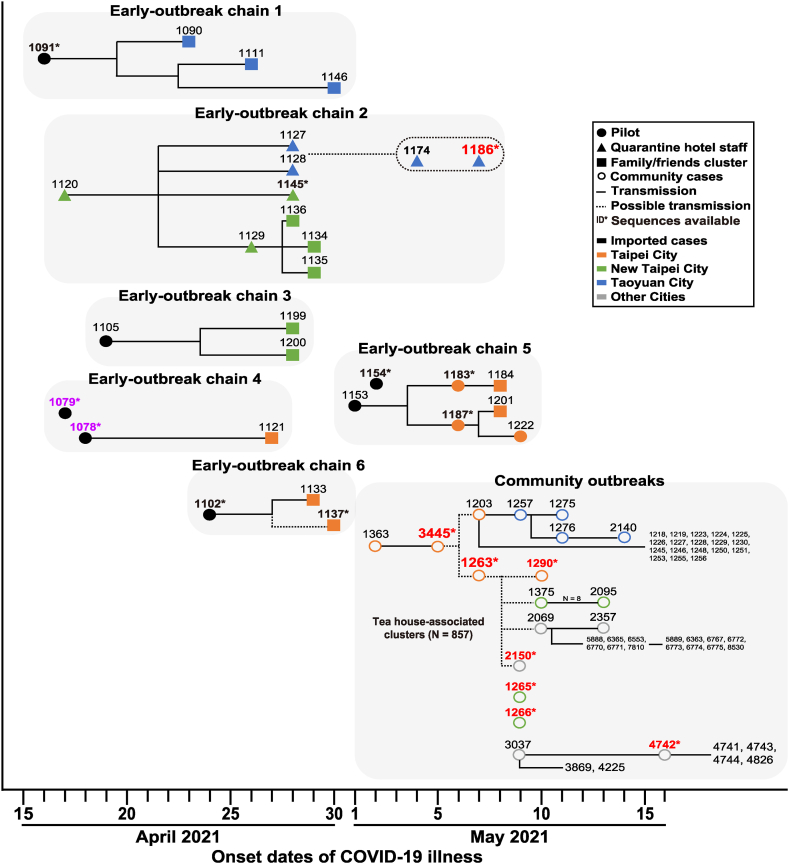
Epidemiological linkages of initial six transmission chains of SARS-CoV-2 cases and their residential districts at the T1 period in the three major affected cities of Taiwan. The initial six Early-outbreak chains were drawn according to Taiwan CDC epidemiological investigations. Symbols and lines shown in each Early-outbreak chain represent the characteristics of the subjects who transmitted the virus (pilot in the circle; hotel staff in the triangle) or new cases from family or friends contacts (in the square) through direct (solid lines) or indirect (dotted lines) transmission. The numbers shown are Case IDs. ID numbers that are red with a star sign have viral sequences available in the GISAID-EpiCoV database.

Given the highly diverse genomic sequences of the Alpha variants and the rapid community spread of the virus in the Taiwan’s 2021 outbreak, it is crucial to understand the possible transmission routes that resulted in large outbreak within a few weeks. Contact-tracing investigations identified six transmission chains prior to the emergence of notable cluster cases led by ID-1363 that ignited the community outbreak (April 16–May 7) (Fig. 4).

**Fig. 5:**
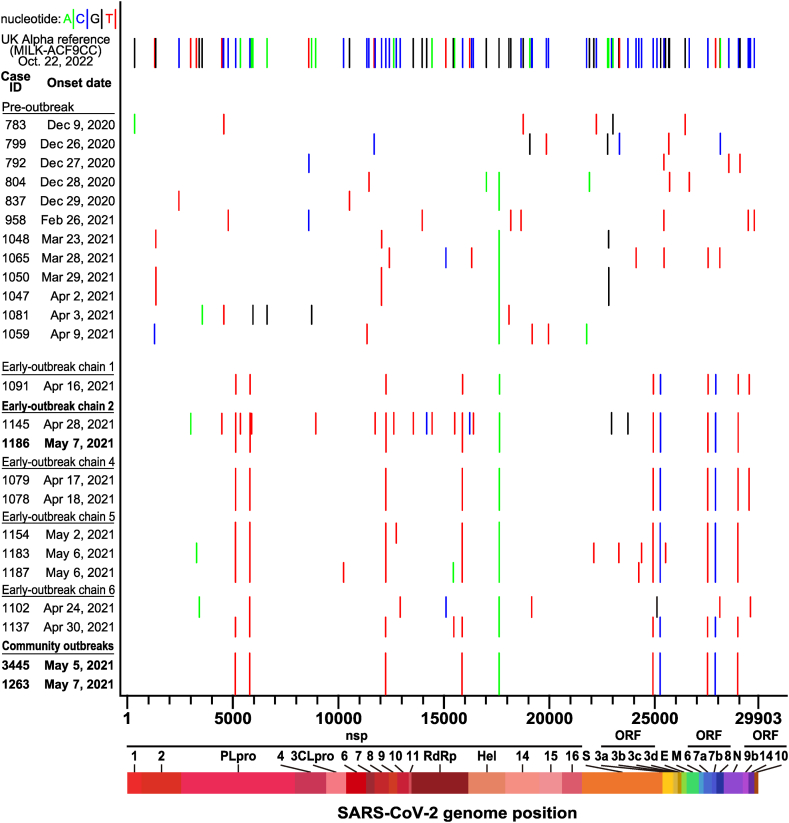
Nucleotide variations of 24 SARS-CoV-2 Alpha variant strains isolated from Pre-outbreak and six different Early-outbreak chains in Taiwan (from December 9, 2020 to May 7) compared to those of the Alpha variant reference strain (UK MILK-ACF9CC). The whole genome sequences of 24 Taiwan SARS-CoV-2 Alpha variant strains were compared to UK-MILK-ACF9CC. The nucleotide variations between Taiwan’s strains and UK-MILK-ACF9CC strain are shown in vertical lines which represent nucleotide A (green), C (blue), G (black), and T (red), respectively.

To delineate which early transmission chain or chains might be attributed to the subsequent community spread in the outbreak, our research integrated whole-genome sequences with contact tracing information for further analysis. We obtained whole-genome sequences of 12 imported viral strains isolated in T0 (pre-outbreak) and 12 strains isolated in T1a (early-outbreak) and found that genomic sequences of the Alpha strains isolated in T0 (before April 16) were highly diverse compared to UK-Alpha-ref-strain. Moreover, they were also different from those imported in T1a (Fig. 5). Comparing with UK-Alpha-ref-strain and the 12 strains in T0, all these early viral strains in T1a contained 6 different common nucleotides (PLpro: C5144T and C5812T, nsp8: C12253T, RdRp: C15895T, Helicase: G17615A, and ORF8: C28957T). However, each of the five different early-outbreak chains [Chain 1 (ID-1091), Chain 2 (ID-1145), Chain 4 (ID-1078 and ID-1079), Chain 5 (ID-1154, ID-1183, ID-1187), and Chain 6 (ID-1102, ID-1137)] had additional nucleotide variations present across the whole-genome (Fig. 5). Noticeably, genomic sequences of ID-1186 isolated from Chain 2 were identical to those sequences of ID-3445 and ID-1263 from the community. Contact-tracing investigations suggested that ID-3445 was a co-worker of the index case ID-1363 at the teahouse. Therefore, we considered ID-3445 and ID-1263 to represent the earliest community-transmission strains in Wanhua District (Fig. 4). Although ID-3445 and ID-1263 had spatially overlapping visiting history, ID-1186 and ID-3445 or ID-1263 had no epidemiolocal-linkage. Moreover, 60% (3/5) of indigenous strains isolated from T1b and 28.57% (16/56) of those isolated from T2 were identical to ID-3445/1263/1186 strain. None of the remaining 42 strains (5 + 56 - 3 - 16 = 42) were identical to any other strains isolated from the early-outbreak clusters.

**Fig. 6:**
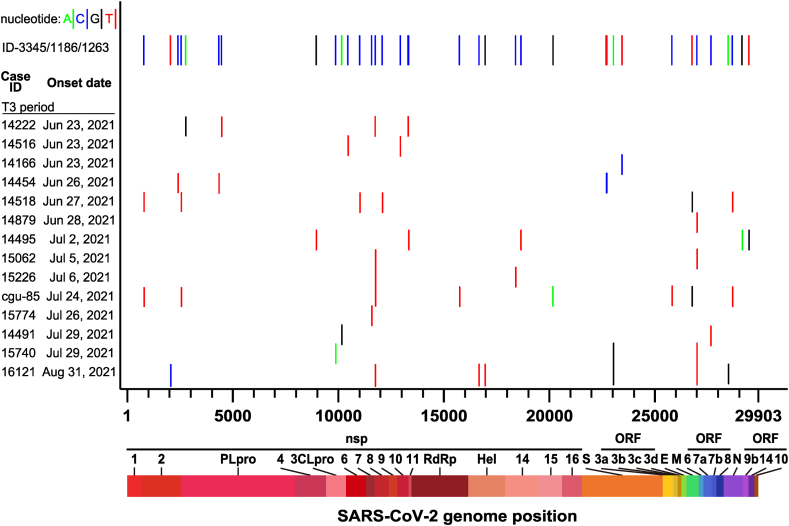
Nucleotide variations of 14 SARS-CoV-2 Alpha variant strains isolated in T3 period compared to the predominant ID-3445/1186/1263 strain. The whole genome sequences of 14 Taiwan SARS-CoV-2 Alpha variant strains isolated in T3 period were compared to ID-3445/1186/1263 strain. The nucleotide variations between T3 strains and T1/T2 predominant ID-3445/1186/1263 strain are shown in vertical lines which represent nucleotide A (green), C (blue), G (black), and T (red), respectively.

These results suggested that despite multiple transmissions that occurred in the early-outbreak period, only the strain from ID-1186 was identical to those of ID-3445 and ID-1263, which were associated with the 2021 community outbreaks (Fig. 4). Interestingly, no other strains identical to the ID-3445/1263/1186 strain were found after the rollout of the Zero-COVID Policy (T3 period). Moreover, whole-genome sequences of all 14 indigenous viral strains isolated during T3 were different and no new dominant virus strain was found. Our analysis did not find a second dominant virus strain throughout the outbreak (Fig. 6).

### 3.4 Epidemiological factors associated with viral strain dominance in the 2021 outbreak

We conducted univariate analysis to identify the factors that were associated with the ID-3445/1263/1186 strains' dominance. Five factors were found to be significant [cOR (95% CI), *p*-value]: (1) epidemic period [1.744 (0.369–7.924), 0.0097], (2) epicenter [0.208 (0.063–0.638), 0.0024], (3) vaccination coverage [0.336 (0.1–1.023), 0.0479], (4) population size [0.219 (0.057–0.789), 0.011], and (5) population density [0.273 (0.086–0.831), 0.018] (Table 1). Among these significant factors, the cOR for T1 had the highest value, indicating that the ID-3445 strain was already predominant in T1. Our multivariable analysis revealed that the epidemic period and epicenter were the two factors significantly associated with the identical-ID-3445/1263/1186 strains’ dominance in the 2021 outbreak [aOR (95% CI), *p*-value: 0.176 (0.05–0.616), 0.007; 0.145 (0.044–0.474), 0.001, respectively] (Table 1, Table S6, Table S7). These epidemiological results (Table 1) integrated with higher viral sequence diversity in T1 (Fig. 5) suggested that the dominant virus strain was selected in the epicenter in T1.

**Table 1:** Univariate analysis and Multivariable regression analysis of the factors associated with the frequency of SARS-CoV-2 genome sequences identical to those of the dominant strains from cluster cases of ID-3445/1186/1263 with their onset dates from December 9, 2020 to October 31, 2021.

			Univariate analysis		Multivariable regression analysis	
Factors	Sequences	Identical to dominant 3445/1186/1263 cluster (%)	Crude OR (95% CI)	*P* value	Adjusted OR (95% CI)	*P* value
**(1) Epidemic periods**						
T1 period	11	45.46% (5/11)	1.74 (0.37–7.92)	0.0097** (T1 vs. T2 = 0.492) (T3 vs. T2 = 0.014*)	**0.18 (0.05**–**0.62)**	0.007**
T2 period	56	32.14% (18/56)	Reference			
T3 period	14	0% (0/14)	0 (0–0.74)			
**(2) Epicenter**						
Epicenter	50	16% (8/50)	0.21 (0.06–0.64)	0.0024**	**0.15 (0.04**–**0.47)**	0.001**
Non-epicenter	31	48.38% (15/31)	Reference			
**(3) Daily city/county-specific vaccination coverage of the 1st-dose COVID-19 (%)**						
<2.87%	41	39.02% (16/41)	Reference	0.0479*	–	–
≥2.87%	40	17.5% (7/40)	0.34 (0.1–1.02)			
**(4) Daily district-specific daily public transport ridership** **(per 10K passengers)**						
<3.344	40	35% (14/40)	Reference	0.225	–	–
≥3.344	41	21.95% (9/41)	0.53 (0.17–1.55)			
**(5) Daily cases**						
<24	26	34.62% (9/26)	Reference	0.435	–	–
≥24	55	25.45% (14/55)	0.65 (0.21–2.04)			
**(6) Monthly district-specific population size (per 100K peoples by district)**						
<1.8	16	56.25% (9/16)	Reference	0.011*	–	–
≥1.8	65	21.54% (14/65)	0.22 (0.06–0.79)			
**(7) Monthly district-specific population density (10K pop. size/district area km**^**2**^**)**						
<2	28	46.43% (13/28)	Reference	0.018*	–	–
≥2	53	18.87% (10/53)	0.27 (0.09–0.83)			
**(8) Age**						
<53	32	25% (8/32)	Reference	1	–	–
≥53	35	25.71% (9/35)	1.04 (0.3–3.65)			
**(9) Gender**						
Female	43	25.58% (11/43)	Reference	0.625	–	–
Male	38	31.58% (12/38)	1.34 (0.46–3.97)			

OR: odds ratio; CI: confidence interval; *P* value: Fisher’s exact test; *: <0.05; **: <0.01.

Multivariable regression formula: binomial linear regression (Identical to ID-3445 = Epidemic periods + Epicenter), AIC = 83.627.

The nine epidemiological factors: (1) epidemic periods (three time periods classified by the major population-based interventions), (2) epicenter (based on the district with the highest incidence rate and its neighboring districts vs others), (3) daily city/county-specific vaccination coverage of the 1st COVID-19 vaccine dose, (4) daily district-specific public transport ridership, (5) daily cases, (6) monthly district-specific population size, (7) monthly district-specific population density, (8) age, and (9) gender.

T1 Period (April 16–May 14; pre-Level 3 Restrictions), T2 Period (May 15–June 22; post-Level 3 Restrictions, but pre-Zero-COVID Policy), and T3 Period (June 23–August 31; post-Zero-COVID Policy) (Supplementary Methods).

We used Fisher’s exact test to assess all factors between subgroups due to the small sample size. Variance inflation factors (VIF >5) were used to evaluate collinearity among factors, and the statistically significant factors without collinearity were included in the final multivariable regression model (Tables S6 and S7).

## 4 Discussion

The fast-mutating and increasingly transmissible SARS-CoV-2 has created unprecedented public health challenges. However, Taiwan successfully halted local SARS-CoV-2 transmission through its rapid response combining strict border control, firm adherence to using facemasks and hand hygiene, and a bundle strategy to minimize nosocomial infection [2]. Alpha variants, which dominated in Europe and the USA in early 2021 [[12], [13], [14]], finally sparked a large outbreak in Taiwan in mid-May 2021 [15]. This study integrated analyses of whole-genome viral sequences with contact-tracing, spatio-temporal analyses, individual-based effective reproductive numbers, and public health policies, to deliver four major findings. First, the Alpha variants introduced to Taiwan were highly diverse. Second, we identified an epicenter Wanhua District in Taipei City, where a convenient transportation hub and many leisure activities facilitated human contacts and viral transmission, driving cases in dense, highly populated neighboring districts, and igniting Taiwan’s large 2021 outbreak. Third, one imported SARS-CoV-2 Alpha variant strain from early-outbreak chains was preferentially selected at the epicenter and became dominant in the early epidemic period. These predominant virus strains extended to the middle period and remained detectable for at least 1.5 months. This was the only dominant virus strain throughout the entire outbreak, but it declined after Level 3 Restrictions were implemented, and became undetectable following the Zero-COVID Policy without city lockdowns [16,17]. Fourth, multivariable regression supported the finding that the early epidemic period and epicenter were significantly associated with emergence of the predominant community-spread viruses. These results indicate the importance of viral genomic surveillance alongside epidemics, and its usefulness in evaluating public health policies.

Given genomic surveillance’s application in control outbreaks [[18], [19], [20], [21]], we linked whole-genome sequencing in Taiwan with epidemiological attributes and discovered that early transmission chains substantially facilitated the mid-April to early May community surge. Therefore, outbreak-associated viral dominance must consider specific epidemiological characteristics [22], including high population density, transportation hubs, and teahouses in the epicenter where patrons mingled without masks, as preludes to this outbreak.

Investigating relationships between epidemiological factors and the emergence, rise, and decline of dominant virus strains is essential for containing outbreaks quickly. In fact, Alpha variants that entered Taiwan before the outbreak had high viral genome divergence. However, after ongoing transmission, virus selection occurred under special epidemiological conditions [8,10,22], like airport-associated cases and earlier community-related clusters [19]. Once the case number sharply rose, indicating the selection-advantageous dominant virus strain was continuously spreading, viral diversity plummeted. As with other VOCs [4], it took 2–3 weeks for community-derived strain to emerge, which became dominant virus strain with more homogeneous genome. Control policies can shape trends in the virus population during this crucial time window. Our data showed that 100 days after the Level 3 Restrictions implementation plus further 61 days following the Zero-COVID Policy’s rollout (Fig. 3), the dominant community-spreading SARS-CoV-2 Alpha strains were successfully eliminated without lockdowns [9,15]. No new dominant virus strain was detected throughout the entire outbreak. Therefore, dominant virus strains with selection advantages must be eliminated quickly before epidemics expand. The highly autonomous and strong public adherence to prevention measures — individual-based precautions such as facemasks and hand hygiene, and population-based intervention policies like minimizing outdoor activities and shuttering social spaces — remains an indispensable element for this successful outbreak control [2,15].

SARS-CoV-2 has continuously evolved worldwide. When the Alpha variant overtook the Wuhan strains [4], it indicated the need to find factors associated with viral dominance. Our multivariable analysis again demonstrated that turning points in the early epidemic period and epicenter supported the emergence of dominant community-spread viruses. This conclusion aligns with our findings on an adaptive mutant in the H1N1pdm09 virus carrying HA2-E374K, which was imported to Taiwan and extended viral survival in a densely populated Taipei City before vaccination rollouts [23]. Our dengue research discovered that clustering dengue cases with higher transmission intensity helped select a virus strain that caused more severe dengue hemorrhagic fever cases in southern Taiwan, where *Aedes aegypti* mosquitoes are assumed to play important roles in viral selection [24,25]. The specific epidemiological conditions for SARS-CoV-2 infection, including human clustering cases, eating and dining without wearing masks, frequent human-to-human contact in entertainment settings (e.g., teahouses), and the combination of low vaccination coverage, and all together helped virus strains with a selective advantage through natural selection (prior to immune selection) become dominant and drive a rapid surge in cases. As these mutants continue evolving, their residues for viral replication, transmissibility, immune antagonism [[26], [27], [28], [29]], and their epidemic or pandemic potential merit monitoring [30].

This study has four major limitations. First, most cases were reported from passive surveillance. Second, we obtained viral sequences retrospectively from databases without random sampling on epidemiological attributes. Many virus strains lacked full-length sequences or complete epidemiological information, resulting in a small sample size and potential selection bias. As we did not have multiple samples from each patient, our results may not fully reflect reality [31,32]. The reproductive numbers of each early transmission chain may be underestimated due to asymptomatic or mild infections. Hence, how early transmission chains and viral selection mechanisms (e.g., for increasing viral infectivity or replication) of dominant virus strains contributed to community clusters remains unclear. Third, individual-based pre-existing comorbidities, vaccination history, past infection, compliance with preventative behavior [2], and other potential influencers of viral dynamics were not collected to protect personal privacy. Fourth, although all 81 indigenous viruses in this outbreak carried Spike-M1237I and Helicase-R460K (Table S5), we still do not know whether or how these mutations might increase viral transmissibility and epidemic severity. However, compiling epidemiological linkages within the same transmission cluster and viral sequences can offer a better picture of early transmission chains.

One important issue is whether we are confident about those low case numbers in the epidemic curve, particularly over Week 20 in 2020 to Week 16 in 2021 (Fig. 2). Due to Taiwan's experience with SARS-CoV in 2003 [1], all pneumonia-like cases, such as severe acute respiratory infections (SARI), mild influenza-like illnesses (ILI), and tuberculosis-like symptoms, are legally required to be reported to the Taiwan CDC. Subsequently, individuals that present with suspected respiratory symptoms must be transferred to specialized isolation wards at medical centers or COVID-19-designated hospitals for laboratory testing, including X-ray examination and specimen collections for PCR confirmation. For those SARS-CoV-2 PCR-positive cases, compulsory contact-tracing and epidemiological investigations were performed to trace each positive case’s footprint. Relatives and anyone who had contact with positive cases at the same place and presented with fever and respiratory symptoms had to be isolated for two weeks. Consequently, Taiwan has detected no community cases for quite a long period (246 days). We are quite confident about the low numbers of symptomatic COVID-19 cases during that period because it is a notifiable disease according to Taiwan's Communicable Disease Control Act. Nevertheless, certain individuals were reluctant to complete two-week quarantines and refused to seek medical attention. Additionally, asymptomatic, or pre-symptomatic community cases, combined with individuals who violate the rules of isolation, escape from quarantine, or avoid other preventive measures, may contribute to the likelihood of, undetected cases in the community.

To achieve more epidemiologically meaningful sample collections, we recommend conducting random sampling on young and old age groups during the early period (when indigenous case numbers are rising versus non-rising) before and after the implementation of different public health policies plus at areas of the epicenter and non-epicenter (defined by high and low incidence rates, respectively).

In summary, Alpha strains in Taiwan started from imported cases with genomic diversity. A dominant virus strain emerged under conditions involving human gatherings, leading to case clusters from the airport to the quarantine hotel, transportation hubs, and teahouses in the epicenter. Four prerequisites for dominant virus strains that possibly emerged in the community include: (1) high frequency of human-to-human contact at hotels without early detection of positive cases, or low compliance with home quarantine, facilitating viral selection without notice, (2) close contacts without adequate protection at teahouses (e.g., removing masks while dining, drinking, or chatting), which may have helped viruses gain selection advantages to increase transmissibility (higher Rt values), (3) highly mobile individuals carrying the virus from the epicenter outward, and (4) lack of effective population-based control policies against continuous transmission, like the initial absence of rapid community screening for SARS-CoV-2-positive cases, low vaccination coverage (1.3% and 0.7% for the 1st dose of the COVID-19 vaccine in Taipei City and New Taipei City as of May 15, 2021, respectively). Importantly, rigorous individual-level and population-level prevention policies on May 15, successfully eliminated the spread of the dominant virus strains. No new viral lineage composition occurred during the 100 days of the 2021 Taiwan outbreak. Future research on VOCs should focus on an integrated approach to timely monitoring of whole-genomic and amino acid changes of novel variants with growing transmissibility, pathogenicity, and fatality, as well as spatio-temporal data analysis to detect dominant virus strains early on.

## 5 Conclusions and prospects

### 5.1 Conclusions

In conclusion, compared to the previously dominant Wuhan strains in Taiwan, the SARS-CoV-2 Alpha pre-dominant strains (i.e. full-length viral sequences identical to the ID-3445 strain) that emerged from the sharply increasing cases in temporal trends and at the epicenter of Taipei City when Taiwan’s large 2021 outbreak began, had a selective advantage in rapid transmission. These dominant virus strains came specifically from the Wanhua District, where population density was high, foot traffic was heavy, and visitors ate and drank without wearing masks in the many local teahouses. These characteristics led the dominant virus strains to spread rapidly from the epicenter to non-epicenter areas [8,9,15]. Such naturally selected dominant virus strain along with low vaccination coverage rates (i.e. before immune selection) could have increased the epidemic’s scale and severity. However, the effective implementation of rigorous individual-based measures, together with population-based intervention strategies such as the Level 3 Restrictions and Enhanced Zero-COVID Policy [15], successfully controlled these dominant virus strains. Unlike in other countries, these measures prevented the dominant virus strains studied here from causing nationwide spread of new SARS-CoV-2 variants, which posed problems elsewhere globally [7]. By integrating and analyzing the full-length SARS-CoV-2 genome sequences and epidemiological factors, our research identified the emergence, rise, and decline of dominant virus strains by analyzing dynamic viral changes across different epidemic periods and areas. These efforts in compiling epidemiological factors, tempo-spatial analyses of viral sequences, bioinformatics, and public health policies, can shed light on the early global containment of fast-spreading dominant virus strains by minimizing cluster cases from facilitating further viral selection.

### 5.2 Practical applications

Our study identified coexistence of Spike-M1237I and the non-structural protein mutation, Helicase-R460K mutations in the dominant virus strain. Hence, practical applications of our study could expand tools for analyzing the correlation between amino acid sequence variations and consensus signatures in both structural and non-structural proteins to promote a more thorough understanding of viral dynamics at different intervention points [33], such as pre- and post-antiviral drug administration, vaccination, and other control measures [34,35]. By unearthing consensus signatures and integrating metadata from interdisciplinary knowledge sharing, we can reveal the dynamic evolution of the virus across different patient cohorts. Also, through structural analysis, our efforts can explore the potential variations and corresponding biological significance of interactions between viral sequence changes and host immunity [36,37]. Further investigation into the evolution of potential immune epitopes could help us better understand about the mechanisms and strategies adopted by SARS-CoV-2 to generate immune escape (e.g., XBB.1.5) [[38], [39], [40]]. In discerning the differences in virus changes between natural selection and vaccine-induced immune selection, we aspire to apply the knowledge and techniques to other emerging pathogens and identify common features between epidemiological characteristics with increasing epidemic and pandemic potential, various prevention and control strategies, and changes in microbial sequences among different strains to support global control efforts.

### 5.3 Future directions

Our results demonstrate that the predominant SARS-CoV-2 virus strains with increasing epidemic and pandemic potential at both the micro- and macro-levels were naturally selected under special epidemiological conditions even before mass vaccination [41]. Furthermore, our software and integrated analyses can be applied to provide timely trends in monitoring full-length viral dynamics, to search for dominant virus strains of any emerging pathogens across an entire epidemic, to identify strains with striking increases in case numbers at an epicenter, as well as evaluate the effectiveness of public health policies [42,43]. In the future, regular analysis of these virus strains through international collaboration can provide reliable insights for genomic surveillance [44]. Continued development of faster multiplexing assays and third-generation sequencing (TGS) technologies will enhance diagnostic and surveillance capabilities for cases presenting either severe acute respiratory infections (SARI) or mild influenza-like illnesses (ILI). For example, conducting regular sentinel surveillance and whole viral genome analysis targeting patients who are at high-risk for COVID-19, such as immunocompromised patients, chronic disease patients, and seniors with comorbidities, even before they develop COVID-like symptoms and signs (i.e. involving asymptomatic or pre-symptomatic high-risk COVID-19 populations to cover possible low-frequency mutants [45]), coupled with specimen collected from patients with respiratory-related illnesses or COVID-19-like symptoms at hospitals, can be more effectively predicting in the dominant virus strains. This integrated analysis is anticipated to provide policymakers with valuable insights for formulating disease prevention policies to reduce the public health threat of any possible selection-advantaged dominant virus strains [38,46].

## Funding statement

This work was supported by grants from the Ministry of Science and Technology (MOST) in Taiwan (MOST-109-2320-B-002-057, MOST-109-2327-B-002-009, and MOST-110-2740-B-002-006) and the National Health Research Institute in Taiwan (NHRI-110A1-MRCO-01212103, NHRI-11A1-MRCO-01222202, and NHRI-12A1-MRCO-01232301). This article was subsidized for English editing by National Taiwan UniversityHospital (NTUH) in Taiwan under the Excellence Improvement Program for Doctoral Students (MOST-108-2926-I-002-002-MY4), sponsored by MOST, Taiwan. None of the funding organizations played any role in the study design, collection, analysis, or interpretation of data, nor in writing the manuscript.

## Data availability statement

All the raw sequence data in this study are available at https://www.gisaid.org (accession number listed in Table S4). The analysis tools, CoVConvert and IniCoV software, are available at https://apps.co-visions.com and https://github.com/chinrur/CoVConvert_IniCoV. In addition, all the methods, as well as Fig. S1 and Tables S1, S2, S3, S4, S5, and S7, are available under “Supplementary Materials.”

## Ethics statement

This study was reviewed and approved by the Research Ethics Committee of National Taiwan University Hospital, with ethics approval reference [Number: 202205059].

## CRediT authorship contribution statement

**Chin-Rur Yang:** Writing – review & editing, Writing – original draft, Visualization, Validation, Software, Resources, Methodology, Investigation, Formal analysis, Data curation. **Sui-Yuan Chang:** Writing – review & editing, Validation, Resources, Data curation. **Yu-Nong Gong:** Writing – review & editing, Validation, Resources. **Chung-Guei Huang:** Writing – review & editing, Validation, Resources. **Tsung-Hua Tung:** Writing – review & editing, Validation, Resources, Investigation, Data curation. **Wei Liu:** Writing – review & editing, Validation, Methodology. **Ta-Chien Chan:** Writing – review & editing, Validation, Methodology. **Kuo-Sheng Hung:** Writing – review & editing, Validation, Resources. **Hung-Sheng Shang:** Writing – review & editing, Validation, Resources. **Jih-Jin Tsai:** Writing – review & editing, Validation, Resources. **Chuan-Liang Kao:** Writing – review & editing, Validation. **Hui-Lin Wu:** Writing – review & editing, Validation. **Li-Yu Daisy Liu:** Writing – review & editing, Validation, Methodology. **Wan-Yu Lin:** Writing – review & editing, Validation. **Yi-Chin Fan:** Writing – review & editing, Validation. **Chwan-Chuen King:** Writing – review & editing, Writing – original draft, Validation, Supervision, Project administration, Funding acquisition, Formal analysis, Conceptualization. **Chia-Chi Ku:** Writing – review & editing, Writing – original draft, Validation, Supervision, Project administration, Funding acquisition, Formal analysis, Conceptualization.

## Declaration of competing interest

The authors declare that they have no known competing financial interests or personal relationships that could have appeared to influence the work reported in this paper.
