# Supplementary material for: The emergence and successful elimination of SARS-CoV-2 dominant strains with increasing epidemic potential in Taiwan’s 2021 outbreak
Source: Heliyon. 2023 Nov 20;9(12):e22436. doi: 10.1016/j.heliyon.2023.e22436 (PMC10724543; doi:10.1016/j.heliyon.2023.e22436)
Supplement: Multimedia component 1 [file mmc1.pdf]

Supplementary Materials

Methods

Analysis of consensus amino acid residues from the collected SARS-CoV-2 strains

The stepwise processes performed by CoVConvert and IniCoV to identify novel signatures of SARS-CoV-2 (Figure S1) were described in our previous study [1].

CoVConvert (Coronavirus viral sequences converter for genome organization) performed virus strains' names, checked the quality of downloaded sequences and achieved multiple alignments (MAFFT 6merpair) [2] based on the Wuhan-Hu-1 reference nucleotide sequence of SARS-CoV-2 (NC045512.2) [3]. Next, data entries with erroneous or incorrect sequences that failed to align were excluded. Lastly, all qualified and well-aligned DNA sequences were translated into three possible polypeptides from 0, + 1, and +2 reading frames to determine one complete full-length viral peptide using CoVConvert (Figure S2).

IniCoV (Coronavirus viral information viewer and analyzer for identifying an initial source), is a program composed of various modules to automatically analyze viral sequencing data in combination with epidemiological information (e.g., viral type, host, region, strain, year, and viral variants or lineages) It comprises the two modules described below [1]:

The CoVCS (Coronavirus Cross-Segment alignment) module was used to align amino acid sequences based on SARS-CoV-2 nomenclature and subsequently divide the translated polypeptides into 31 proteins as illustrated in Figure S2. CoVCS-processed viral genetic information can easily be used to determine the sequence and genome organization by Transcription-regulatory sequences (TRS) (intergenic sequence) [4, 5] based on a particular residue [1].

The CoVCG (Coronavirus Comparative Grouping) module was designed to automatically deduce amino acid sequences from the collected SARS-CoV-2 strains

grouped by the question of interest as described in our previous study [1]. In short, CoVCG first generated consensus sequences from each subgroup and determined the most representative (i.e., most frequent) amino acid at each position through computing. Unique amino acid residues differentially presented between different subgroups in the whole genome of SARS-CoV-2 computed by CoVCG were re-examined, verified based on the CoVCG-generated substitution table, and visualized.

The packages in the R programming language

We used the following four packages for statistical analysis in this study, according to the user manuals in the Comprehensive R Archive Network (CRAN; https://cran.r-project.org/) descriptions:

(1) Analyze Overdispersed Data (aod): provides a set of functions to Analyze Overdispersed Data (AOD; counts or proportions). The functions should be considered complements to more sophisticated methods such as generalized linear models (GLM).

(2) Modern Applied Statistics with S (MASS): Support Functions and Datasets for Venables and Ripley's "Modern Applied Statistics with S". (4th edition, 2002).

(3) Companion to Applied Regression (car): J. Fox and S. Weisberg, an R Companion to Applied Regression, Third Edition, Sage, 2019.

(4) R Commander (Rcmdr): a platform-independent basic-statistics GUI (graphical user interface) for R, and provided multiple useful tools for statistical (e.g. generalized linear models (GLM), and stepwise regression).

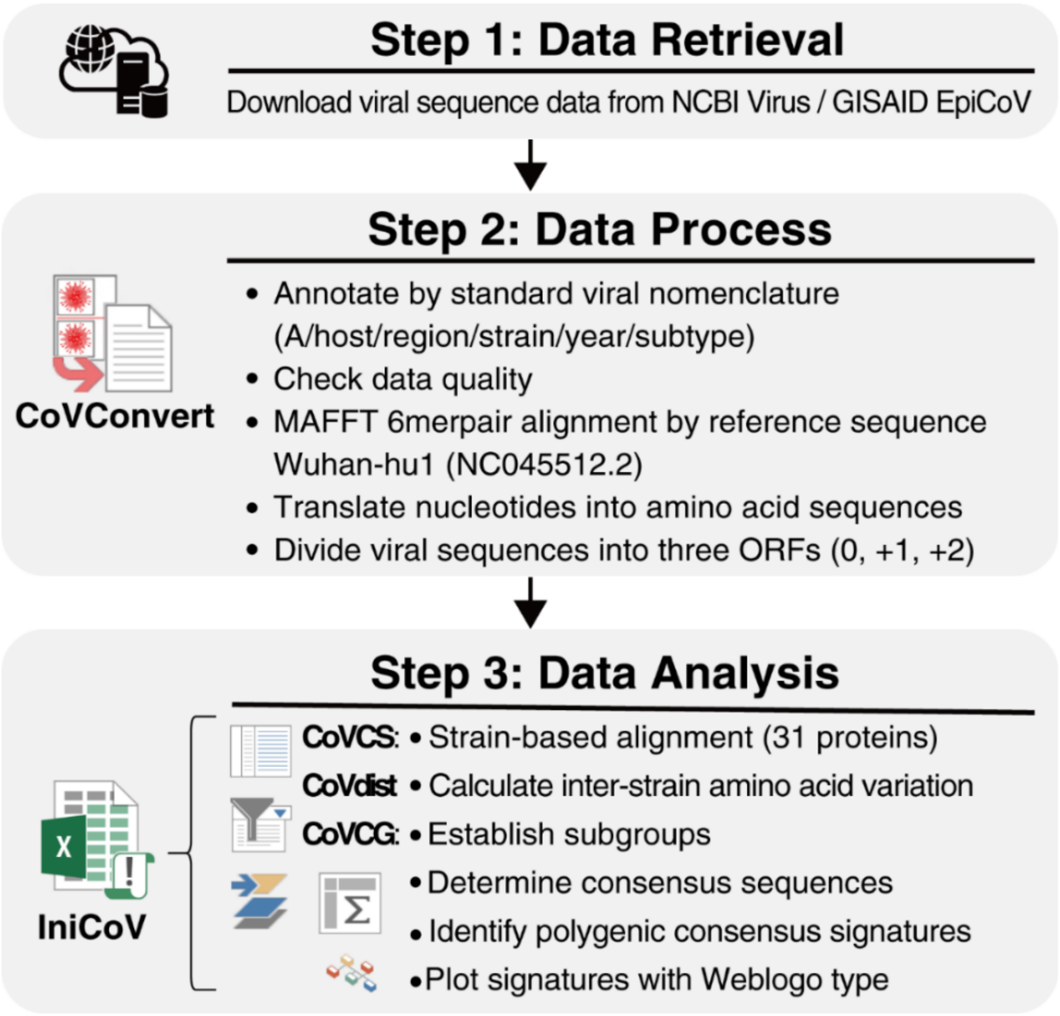

Figure S1. Workflows of data analysis executed by CoVConvert and IniCoV

The stepwise processes performed by CoVConvert and IniCoV to identify novel signatures of SARS-CoV-2 are as follow:

Step 1: Viral sequences are obtained from NCBI-Virus and GISAID-EpiCoV databases.

Step 2: CoVConvert rearranges viral strains and confirms data quality. Viral sequences are further sorted into three ORFs and translated into 31 protein sequences.

Step 3: The CoVCS module of IniCoV organize CoVConvert-processed viral amino acid sequences. The CoVCG module of IniCoV regroups viral strains with epidemiological significance and computes the highest frequency of each residue to generate a consensus sequence for each subgroup.

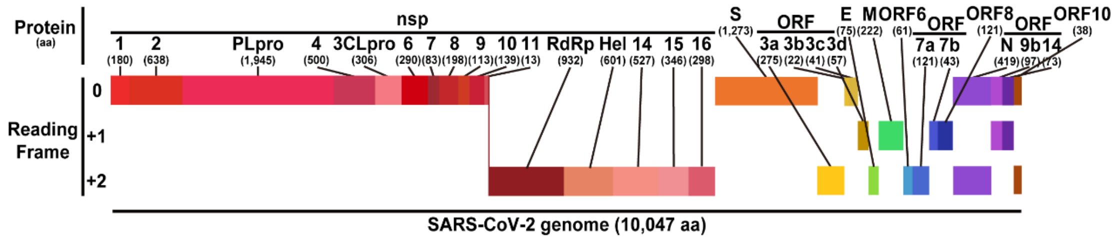

Figure S2. Schematic of 31 proteins on genome organization of SARS-CoV-2

SARS-CoV-2 is a single-stranded RNA virus containing 10,047 amino acids (~30 kb genome size); 67% of the genome encodes 16 nonstructural proteins (nsp), and the remaining 33% of the genome encodes 15 structural proteins. In structural proteins, when more ORFs are expressed from the same short genomic RNA (sgRNA), these are called a-d (e.g., ORF3a, ORF3c) [6]. Blocks in different colors represent 31 different protein sequences: the size corresponds to the amino acid residues of the sequences, and the position shows the possible polypeptides from 0, +1, +2 reading frames.

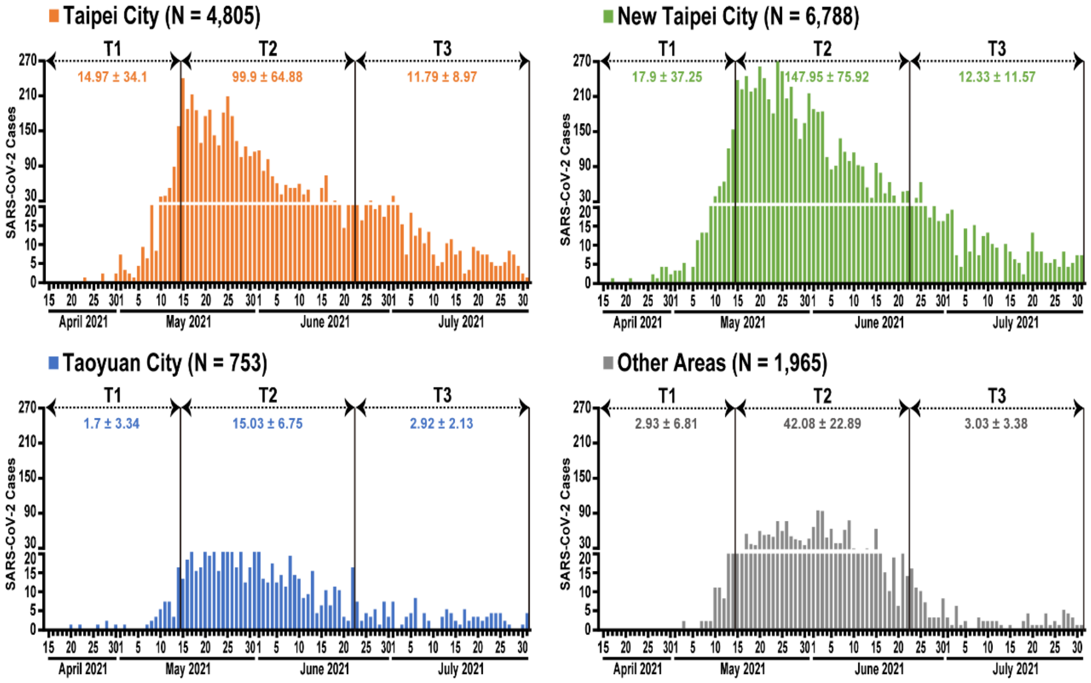

Figure S3. Epidemic curves of laboratory-confirmed SARS-CoV-2 cases in the three major affected cities and other areas of Taiwan in the large 2021 outbreak

The bar graphs show the distributions of cases based on onset dates from April 15 through July 31, 2021. Because the daily numbers of confirmed SARS-CoV-2 cases in Taoyuan City were much lower than those in Taipei and New Taipei cities, we used two scales (0-20 and 30-270 cases) that are separated by white lines in Taipei City, New Taipei City, and other areas.

The 81 indigenous strains involved three time periods based on population-based interventions: 1) T1 Period (April 15-May 14; pre-Level 3 restrictions), 2) T2 Period (May 15-June 21; post-Level 3 restrictions, but pre-Zero COVID policy), and 3) T3 Period (June 23-August 31; post-Zero COVID policy).

The mean weekly numbers are shown as “mean ± SD” before and after the 2021 outbreak, Taipei City: 0.516 ± 1.807 vs 1201.25 ± 1372.857 ($p < 0.0001$), New Taipei City: 0.516 ± 2.38 vs 1939.429 ± 1975.374 ($p < 0.0001$), and Taoyuan City: 2.323 ± 5.11 vs 215.143 ± 172.747 ($p < 0.0001$).

Table S1. List of 24 SARS-CoV-2 Alpha variant cases from three risk-clusters in the onset of 2021 large outbreak in Taiwan (from December 9, 2020 to May 16, 2021)

Case ID	Onset date	Imported/ Indigenous	Location	Cluster	Age	Gender	Helicase R460K	Spike M1237I	Epidemiologic linkage (Case ID)	Rt+
783	December 9, 2020	Imported	Philippines	-	27	M	R	M	NA	0
799	December 26, 2020	Imported	UK	-	75	M	R	M	NA	0
792	December 27, 2020	Imported	UK	-	20	M	R	M	NA	0
804	December 28, 2020	Imported	UK	-	37	M	K	M	NA	0
837	December 29, 2020	Imported	UK	-	32	M	K	M	NA	0
958	Feburary 26, 2021	Imported	USA	-	52	M	R	M	NA	0
1048	March 23, 2021	Imported	Philippines	-	63	M	K	M	NA	0
1065	March 28, 2021	Imported	Philippines	-	32	M	K	M	NA	0
1050	March 29, 2021	Imported	Egypt	-	20	M	K	M	NA	0
1047	March 29, 2021	Imported	Indonesia	-	23	M	K	M	NA	0
1081	March 10, 2021	Imported	Indonesia	-	41	M	K	M	NA	0
1059	April 9, 2021	Imported	Japan	-	24	M	K	M	NA	0
1091	April 16, 2021	Imported	USA	pilot	52	M	K	I	1090, 1111, 1146	3
1105	April 19, 2021	Imported	USA	pilot	46	M	NA	NA	1199, 1200	2
1078	April 18, 2021	Imported	USA	pilot	52	M	K	I	1121	1
1153	May 1, 2021	Imported	USA	pilot	37	M	K	I	1183, 1187	2
1102	April 24, 2021	Imported	USA	pilot	38	M	K	M	1133, 1137	2
1120	April 17, 2021	Indigenous	New Taipei City	hotel staff	48	M	NA	NA	1127, 1128, 1129, 1145	4
1363	May 2, 2021	Indigenous	Taipei City	community	62	M	NA	NA	**Earliest Wanhua case and transmitted to 3445**	1
3445	May 5, 2021	Indigenous	Taipei City	community	53	F	K	I	4008, 4009, 4010, 4216, 4305	5
1203	May 7, 2021	Indigenous	Taipei City	community	64	M	NA	I	1218, 1219, 1223, 1224, 1225, 1226, 1227, 1228, 1229, 1230, 1245, 1246, 1248, 1250, 1251, 1253, 1255, 1256, 1257	19
1257	May 9, 2021	Indigenous	Taoyuan City	1203's family	47	M	NA	NA	1275, 1276, 2140	3
3037	May 9, 2021	Indigenous	Pingtung County	Community (Wanhua travel history)	65	M	NA	NA	3869, 4225, 4742, 4743	4
4742	May 16, 2021	Indigenous	Kaohsiung City	3037's family	56	M	K	I	4741, 4743, 4744, 4826	4

*ID-1363, 3445, and 1203 had visited the same tea house in the Wanhua District.

The mean ± SD of Rt (Reproductive number over time values) values: the five pilots (onset dates from 16 April to 1 May 2021) associated clusters was 2 ± 0.71 (range 1-3), one hotel-staff (onset date on 17 April 2021) associated cluster was 4, and six earlier community-associated clusters (onset dates for the first case of each cluster ranged from 2 May to 16 May 2021) was 6 ± 6.51 (range 1-19), p = 0.007 (One-way ANOVA)

+Rt values of the 3 types of risk-sources for the early outbreak:

1) The pilot-associated clusters (ID-1091, 1078, 1105, 1102, and 1153): mean ± SD value of Rt = 2 [(3+1+2+2+2)/5]

2) The quarantine hotel employee-associated cluster (ID-1120): Rt = 4

3) The early community-associated clusters (ID-3445, 1203, 1257, 3037, 4742): mean ± SD value of Rt = 7 ± 6.75 [(5+19+3+4+4)/5]

Table S2. The district-specific incidence rates of the SARS-CoV-2-positive cases, population sizes, and population densities in the three affected cities by the four time periods during Taiwan 2021 large outbreak

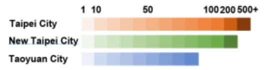

Districts in the three outbreak affected cities	Incidence Rates (per 100K)				Population Size	Population Density	Area
	T1a	T1b	T2	T3			
	4/16~5/6	5/7~5/14	5/15~6/22	6/23~7/31	(May 2021)	(Size/Area)	(km^2)
**Wanhua District, Taipei City**	9.42	129.16	810.25	86.05	180,396	20,378.66	8.85
**Zhonghe District, New Taipei City***	1.46	17.09	241.03	12.70	409,649	20,336.03	20.14
**Banqiao District, New Taipei City***	0.54	24.09	209.69	14.75	556,175	24,038.03	23.14
**Datong District, Taipei City***	0.81	22.75	204.00	14.63	123,085	21,664.17	5.68
**Yonghe District, New Taipei City***	2.74	15.09	199.56	14.20	218,652	38,267.35	5.71
**Sanchong District, New Taipei City***	2.87	15.89	181.12	23.20	383,805	23,521.79	16.32
**Zhongzheng District, Taipei City***	1.30	12.98	154.57	15.60	154,098	20,257.13	7.61
Tucheng District, New Taipei City	1.26	13.44	179.35	10.50	238,114	8,055.88	29.56
Wugu District, New Taipei City		17.81	150.19	24.48	89,842	2,576.99	34.86
Luzhou District, New Taipei City	2.47	12.35	124.04	10.38	202,410	27,223.57	7.44
Shilin District, Taipei City		3.63	126.55	17.09	275,204	4,412.57	62.37
Shiding District, New Taipei City		13.33	133.32		7,503	51.98	144.35
Xinzhuang District, New Taipei City		8.98	122.48	11.11	422,978	21,429.30	19.74
Wenshan District, Taipei City	1.13	7.52	116.26	13.17	265,885	8,438.38	31.51
Xinyi District, Taipei City	0.94	8.49	107.64	17.01	211,920	18,908.43	11.21
Taishan District, New Taipei City	1.28	7.69	106.41	16.67	78,010	4,071.44	19.16
Zhongshan District, Taipei City	0.45	5.43	103.71	19.03	220,944	16,148.40	13.68
Shenkeng District, New Taipei City		8.41	88.35	29.46	23,774	1,155.27	20.58
Xindian District, New Taipei City	0.33	9.26	103.19	11.57	302,503	2,516.13	120.23
Nangang District, Taipei City	1.70	9.35	99.53	5.95	117,606	5,384.30	21.84
Shulin District, New Taipei City	0.55	8.75	89.70	5.47	182,849	5,519.34	33.13
Xizhi District, New Taipei City	0.49	2.92	90.41	7.29	205,812	2,889.18	71.24
Daan District, Taipei City	0.67	8.03	79.08	10.06	298,891	26,307.59	11.36
Beitou District, Taipei City		4.83	79.81	9.67	248,237	4,368.71	56.82
Jinshan District, New Taipei City		4.77	85.86		20,977	426.25	49.21
Songshan District, Taipei City		8.08	73.23	9.09	198,120	21,331.21	9.29
Guishan District, Taoyuan City	0.61	4.84	75.60	6.04	165,261	2,294.73	72.02
Bali District, New Taipei City		5.03	77.96	2.51	39,734	1,006.09	39.49
Tamsui District, New Taipei City	1.09	7.06	64.59	8.14	184,240	2,607.54	70.66
Sanxia District, New Taipei City		5.14	65.99	5.14	116,708	609.60	191.45
Linkou District, New Taipei City		2.44	63.52	5.70	122,792	2,267.55	54.15
Neihu District, Taipei City	0.36	4.99	48.53	8.57	280,318	8,876.81	31.58
Taoyuan District, Taoyuan City	0.22	2.84	38.39	5.89	458,376	13,169.98	34.80
Daxi District, Taoyuan City			31.48	10.49	95,276	906.35	105.12
Shimen District, New Taipei City			35.29		11,353	221.46	51.26
Yingge District, New Taipei City		1.14	31.86	1.14	87,850	4,158.62	21.12
Ruifang District, New Taipei City			33.48		38,839	549.09	70.73
Bade District, Taoyuan City		2.39	24.36	6.69	209,290	6,208.34	33.71
Wulai District, New Taipei City				31.67	6,315	19.66	321.13
Sanzhi District, New Taipei City			31.15		22,487	340.76	65.99
Luzhu District, Taoyuan City	2.40	2.40	19.19	4.20	166,744	2,208.46	75.50
Wanli District, New Taipei City		13.92	13.92		21,555	340.11	63.38
Zhongli District, Taoyuan City	0.71	1.18	18.69	5.44	422,582	5,522.50	76.52
Longtan District, Taoyuan City		0.80	15.27	8.04	124,368	1,653.08	75.23
Dayuan District, Taoyuan City	1.07	1.07	15.99	5.33	93,814	1,073.48	87.39
Yangmei District, Taoyuan City		0.57	14.21	0.57	175,836	1,972.96	89.12
Pinglin District, New Taipei City			14.95		6,688	39.15	170.84
Pingzhen District, Taoyuan City		2.19	10.50	2.19	228,594	4,786.99	47.75
Guanyin District, Taoyuan City			11.56	1.44	69,211	786.66	87.98
Xinwu District, Taoyuan City			4.06	2.03	49,218	578.92	85.02

Rankings of top district-specific incidence rates of SARS-CoV-2 in the three affected cities (Taipei, New Taipei, and Taoyuan cities) during the large 2021 outbreak (April 16 – July 31, 2021) in Taiwan. The Wanhua District had a 3.28-5.42-fold higher incidence than the next highest-ranking district.

* Distances to the center of the six districts in the three affected cities close to the Wanhua District were 2 km, 2.5 km, 3.5 km, 3.5 km, 3.9 km, and 4.1 km in the Zhongzheng, Yonghe, Zhonghe, Datong, Banqiao, and Sanchong Districts respectively.

Table S3. The number of nucleotide variations of the 81 indigenous SARS-CoV-2 Alpha variant strains in Taiwan compared to the Alpha reference strains (UK-MILK-ACF9CC) in the three time periods

	No. of strains	No. of SNV (mean ± SD)	% SNV (mean ± SD)	P value (Period vs. all)
T1 period April 16-May 14	11	12.36 ± 4.18	0.0413 ± 0.014	0.4954
T2 period May 15- June 22	56	11.29 ± 1.44	0.0377 ± 0.0048	0.135
T3 period June 23- August 31	14	13.43 ± 2.31	0.0449 ± 0.0077	0.0154*

SNV: single nucleotide variation

P value: Student's t-test; *: <0.05.

Table S4-1. List of 101 SARS-CoV-2 genome sequences and important epidemiological information used in this study in Taiwan

Case ID	Strain Name	Onset date	Travel history	Identical to ID-3445	GISAID Accession (EPI_ISL)
**T0 Imported cases (N = 12)**					
783	cgmh-cgu-44	2020/12/9	PHL	-	956325
799	ntu52	2020/12/26	GBR	-	1041958
792	792	2020/12/27	GBR	-	1381386
804	ntu49	2020/12/28	GBR	-	1010728
837	ntu54	2020/12/29	GBR	-	1039160
958	cgmh-cgu-58	2021/2/26	USA	-	2249597
1048	cgmh-cgu-61	2021/3/23	PHL	-	2250151
1065	ntu62	2021/3/28	PHL	-	1667475
1050	ntu61	2021/3/29	EGY	-	1667474
1047	cgmh-cgu-60	2021/4/2	IDN	-	2249836
1081	cgmh-cgu-63	2021/4/3	IDN	-	2250184
1059	kmuh-3	2021/4/9	JPN	-	5395633
**T1 Imported cases (N = 8)**					
1091	ntu63	2021/4/16	USA	-	13566006
1079	1079	2021/4/17	USA	-	2455264
1078	1078	2021/4/18	USA	-	2455327
1102	ntu64	2021/4/24	USA	-	15971077
1144	ntu65	2021/4/28	UZB	-	-
1154	ntu67	2021/5/2	USA	-	13618360
1183	tsgh-43	2021/5/6	USA	-	2693006
2018	cgmh-cgu-64	2021/5/14	HTI	-	2544700
**T1 Epicenter (N = 9)**					
1145	tsgh-42	2021/4/28	NWT	-	2693005
1137	tsgh-44	2021/4/30	TPE	-	4096803
3445	3445	2021/5/5	TPE	Yes	2455329
1187	ntu66	2021/5/6	TPE	No	13618344
1263	ntu68	2021/5/7	TPE	Yes	13578728
1266	ntu69	2021/5/9	NWT	No	13578729
1265	ntu70	2021/5/9	NWT	No	13578730
1290	ntu71	2021/5/10	TPE	No	13578731
2262	2262	2021/5/14	TPE	Yes	2455330
1145	tsgh-42	2021/4/28	NWT	-	2693005
**T1 Other cities (N = 2)**					
1186	cgmh-cgu-73	2021/5/7	TAO	Yes	2544709
2150	kmuh-4	2021/5/9	KHH	Yes	7016374
**T2 Epicenter (N = 30)**					
1419	ntu72	2021/5/15	TPE	No	13578732
1373	ntu73	2021/5/15	TPE	Yes	13578733
1354	ntu74	2021/5/15	TPE	No	13618345
1359	ntu75	2021/5/15	TPE	No	13578734
1356	ntu76	2021/5/15	TPE	No	13578345
1357	ntu77	2021/5/15	TPE	Yes	13618347
1355	ntu78	2021/5/15	TPE	No	13578735
1360	ntu79	2021/5/15	TPE	No	13578736
1358	ntu80	2021/5/15	TPE	No	13578737
5703	5703	2021/5/21	TPE	No	3000790

Table S4-2. List of 101 SARS-CoV-2 genome sequences and important epidemiological information used in this study in Taiwan *(continued)*

Case ID	Strain Name	Onset date	Travel history	Identical to ID-3445	GISAID Accession (EPI_ISL)
**T2 Epicenter (N = 30)**					
7955	7955	2021/5/26	TPE	No	3040151
9098	9098	2021/5/29	NWT	Yes	3040149
10747	10747	2021/6/2	TPE	No	3000409
12049	ntu94	2021/6/11	TPE	No	11333413
10179	ntu91	2021/6/12	TPE	No	11333514
13112	ntu95	2021/6/12	TPE	No	11333432
13375	13375	2021/6/14	TPE	No	3001055
13435	13435	2021/6/14	TPE	No	3040140
13564	13564	2021/6/15	TPE	No	3001368
11612	ntu81	2021/6/16	TPE	No	13578738
13137	ntu104	2021/6/16	TPE	No	11333509
13386	ntu82	2021/6/17	TPE	No	13578739
13103	ntu83	2021/6/17	TPE	No	13618348
13318	ntu84	2021/6/17	TPE	No	13578740
10480	ntu85	2021/6/17	TPE	No	13578741
13387	ntu88	2021/6/18	TPE	Yes	11333411
13850	ntu107	2021/6/19	TPE	Yes	11333511
14035	ntu98	2021/6/20	TPE	No	11333516
14168	ntu105	2021/6/20	TPE	No	11333510
14181	ntu108	2021/6/21	TPE	No	11333512
**T2 Other cities (N = 26)**					
3461	kmuh-5	2021/5/16	KHH	No	7016459
4742	kmuh-6	2021/5/16	KHH	No	7016494
-	cgmh-cgu-65	2021/5/18	TAO	Yes	2544701
-	cgmh-cgu-66	2021/5/18	TAO	Yes	2544702
-	cgmh-cgu-79	2021/5/18	TAO	No	5160472
-	cgmh-cgu-68	2021/5/19	TAO	Yes	2544704
-	cgmh-cgu-67	2021/5/20	TAO	No	2544703
-	cgmh-cgu-70	2021/5/20	TAO	Yes	2544706
-	cgmh-cgu-76	2021/5/20	TAO	Yes	2544712
-	cgmh-cgu-69	2021/5/21	TAO	No	2544705
-	cgmh-cgu-78	2021/5/22	TAO	Yes	2544714
-	cgmh-cgu-77	2021/5/23	TAO	No	2544713
-	cgmh-cgu-75	2021/5/26	TAO	No	2544711
-	cgmh-cgu-72	2021/5/27	TAO	No	2544708
-	cgmh-cgu-74	2021/5/29	TAO	No	2544710
10321	10321	2021/6/1	MIA	Yes	3040148
11042	11042	2021/6/3	TAO	Yes	3040145
11103	11103	2021/6/3	CYQ	Yes	3040147
11102	11102	2021/6/3	TNN	No	3040152
11310	11310	2021/6/4	MIA	Yes	3040146
11282	tsgh-46	2021/6/4	KEE	No	4096807
12288	12288	2021/6/8	TAO	Yes	3040144
12857	12857	2021/6/10	KEE	Yes	3001841
12699	12699	2021/6/10	KEE	No	3002178
12828	12828	2021/6/10	TAO	No	3040141
14222	14222	2021/6/20	KEE	Yes	3040143

Table S4-3. List of 101 SARS-CoV-2 genome sequences and important epidemiological information used in this study in Taiwan *(continued)*

Case ID	Strain Name	Onset date	Travel history	Identical to ID-3445	GISAID Accession (EPI_ISL)
**T3 Epicenter (N = 11)**					
14422	ntu101	2021/6/23	TPE	No	11333507
14516	ntu102	2021/6/23	TPE	No	11333508
14166	ntu106	2021/6/23	TPE	No	11362237
14518	ntu116	2021/6/27	TPE	No	11333513
14879	ntu113	2021/6/28	TPE	No	11362240
14495	ntu103	2021/7/2	TPE	No	11333517
15062	ntu111	2021/7/5	TPE	No	11362238
15226	ntu117	2021/7/6	TPE	No	11362241
15774	tsgh-45	2021/7/28	TPE	No	4096805
15702	ntu123	2021/7/29	TPE	No	11362244
16121	ntu124	2021/8/31	TPE	No	11333351
**T3 other cities (N = 3)**					
14491	kmuh-7	2021/6/23	KHH	No	7016498
14454	14454	2021/6/26	MIA	No	3040142
-	cgmh-cgu-85	2021/7/24	TAO	No	5160564

EGY: Egypt, GBR: United Kingdom, HTI: Haiti, IDN: Indonesia, JPN: Japan, PHL: Philippines, UZB: Uzbekistan.

CYQ: Chiayi City, KEE: Keelung City, KHH: Kaohsiung City, MIA: Miaoli County, NWT: New Taipei City, TAO: Taoyuan City, TNN: Tainan City, TPE: Taipei City.

NTU: National Taiwan University, Taiwan CDC: Taiwan Centers for Disease Control, TSGH: Tri-Service General Hospital, CGMH-CGU: Chang Gung Memorial Hospital (University), KMUH: Kaohsiung Medical University Chung-Ho Memorial Hospital.

We used 101 available Taiwan whole-genome sequences of SARS-CoV-2 for analysis. Imported or Indigenous cases were defined through joint epidemiological investigation efforts from local Health Bureaus and Taiwan CDC. A case that had travel history was defined as an imported case.

Table S5. Mutation prevalence percentages of the 101 Taiwan Alpha variant strains compared to those of the Alpha variant reference strain (UK-MILK-ACF9CC)

Residue		Reference Prevalence (%)	No. of Strains		Mutation Prevalence (%)	No. of Strains
Hel_460	R	3.96%	4	**K**	**96.04%**	**97**
S_1237	M	13.86%	14	**I**	**86.14%**	**87**
nsp6_260	L	89.11%	90	**F**	**10.89%**	**11**
M_82	I	94.06%	95	**S/T**	**5.94%**	**6**
nsp1_170	T	95.05%	96	**I**	**4.95%**	**5**
N_135	T	95.05%	96	**I**	**4.95%**	**5**
nsp2_169	L	97.03%	98	**F**	**2.97%**	**3**
N_398	A	97.03%	98	**V**	**2.97%**	**3**
nsp4_17	F	98.02%	99	**L**	**1.98%**	**2**
3CLpro_160	C	98.02%	99	**F**	**1.98%**	**2**
nsp8_141	T	98.02%	99	**M**	**1.98%**	**2**
nsp9_83	P	98.02%	99	**L**	**1.98%**	**2**
RdRp_671	G	98.02%	99	**S**	**1.98%**	**2**
nsp15_185	V	98.02%	99	**I**	**1.98%**	**2**
S_69	-	98.02%	99	**H**	**1.98%**	**2**
S_70	-	98.02%	99	**V**	**1.98%**	**2**
S_144	-	98.02%	99	**Y**	**1.98%**	**2**
ORF3a_15	L	98.02%	99	**F**	**1.98%**	**2**
ORF7a_96	L	98.02%	99	**F**	**1.98%**	**2**
ORF8_27	X	98.02%	99	**Q**	**1.98%**	**2**
ORF8_68	K	98.02%	99	**-**	**1.98%**	**2**

Residue		Reference Prevalence (%)	No. of Strains		Mutation Prevalence (%)	No. of Strains	Residue		Reference Prevalence (%)	No. of Strains		Mutation Prevalence (%)	No. of Strains
nsp1_29	R	99.01%	100	**H**	**0.99%**	**1**	S_62	V	99.01%	100	**L**	**0.99%**	**1**
nsp1_104	L	99.01%	100	**I**	**0.99%**	**1**	S_142	G	99.01%	100	**D**	**0.99%**	**1**
nsp2_163	F	99.01%	100	**L**	**0.99%**	**1**	S_145	Y	99.01%	100	**-**	**0.99%**	**1**
nsp2_261	E	99.01%	100	**D**	**0.99%**	**1**	S_156	E	99.01%	100	**G**	**0.99%**	**1**
nsp2_430	S	99.01%	100	**L**	**0.99%**	**1**	S_157	F	99.01%	100	**-**	**0.99%**	**1**
nsp2_528	T	99.01%	100	**I**	**0.99%**	**1**	S_158	R	99.01%	100	**-**	**0.99%**	**1**
nsp2_550	L	99.01%	100	**F**	**0.99%**	**1**	S_181	G	99.01%	100	**V**	**0.99%**	**1**
nsp2_552	P	99.01%	100	**S**	**0.99%**	**1**	S_221	S	99.01%	100	**L**	**0.99%**	**1**
PLpro_13	I	99.01%	100	**V**	**0.99%**	**1**	S_222	A	99.01%	100	**V**	**0.99%**	**1**
PLpro_96	F	99.01%	100	**L**	**0.99%**	**1**	S_224	E	99.01%	100	**Q**	**0.99%**	**1**
PLpro_183	I	99.01%	100	**T**	**0.99%**	**1**	S_385	T	99.01%	100	**N**	**0.99%**	**1**
PLpro_231	A	99.01%	100	**T**	**0.99%**	**1**	S_452	L	99.01%	100	**R**	**0.99%**	**1**
PLpro_277	G	99.01%	100	**E**	**0.99%**	**1**	S_463	P	99.01%	100	**A**	**0.99%**	**1**
PLpro_522	E	99.01%	100	**K**	**0.99%**	**1**	S_478	T	99.01%	100	**K**	**0.99%**	**1**
PLpro_689	L	99.01%	100	**F**	**0.99%**	**1**	S_484	E	99.01%	100	**G**	**0.99%**	**1**
PLpro_724	T	99.01%	100	**I**	**0.99%**	**1**	S_493	Q	99.01%	100	**R**	**0.99%**	**1**
PLpro_822	P	99.01%	100	**L**	**0.99%**	**1**	S_501	Y	99.01%	100	**N**	**0.99%**	**1**
PLpro_881	Y	99.01%	100	**F**	**0.99%**	**1**	S_570	D	99.01%	100	**A**	**0.99%**	**1**
PLpro_890	D	99.01%	100	**A**	**0.99%**	**1**	S_577	R	99.01%	100	**L**	**0.99%**	**1**
PLpro_1079	D	99.01%	100	**G**	**0.99%**	**1**	S_681	H	99.01%	100	**R**	**0.99%**	**1**
PLpro_1302	K	99.01%	100	**R**	**0.99%**	**1**	S_716	I	99.01%	100	**T**	**0.99%**	**1**
PLpro_1412	T	99.01%	100	**I**	**0.99%**	**1**	S_719	T	99.01%	100	**S**	**0.99%**	**1**
nsp4_130	G	99.01%	100	**V**	**0.99%**	**1**	S_892	A	99.01%	100	**V**	**0.99%**	**1**
nsp4_163	S	99.01%	100	**F**	**0.99%**	**1**	S_950	D	99.01%	100	**N**	**0.99%**	**1**
nsp4_439	T	99.01%	100	**K**	**0.99%**	**1**	S_982	A	99.01%	100	**S**	**0.99%**	**1**
nsp4_446	A	99.01%	100	**V**	**0.99%**	**1**	S_1118	H	99.01%	100	**D**	**0.99%**	**1**
3CLpro_274	N	99.01%	100	**S**	**0.99%**	**1**	S_1146	D	99.01%	100	**H**	**0.99%**	**1**
nsp6_106	-	99.01%	100	**S**	**0.99%**	**1**	S_1191	K	99.01%	100	**T**	**0.99%**	**1**
nsp6_107	-	99.01%	100	**G**	**0.99%**	**1**	ORF3a_12	T	99.01%	100	**I**	**0.99%**	**1**
nsp6_108	-	99.01%	100	**F**	**0.99%**	**1**	ORF3a_26	S	99.01%	100	**L**	**0.99%**	**1**
nsp6_128	A	99.01%	100	**V**	**0.99%**	**1**	ORF3a_39	A	99.01%	100	**S**	**0.99%**	**1**
nsp6_149	V	99.01%	100	**A**	**0.99%**	**1**	ORF3a_90	V	99.01%	100	**F**	**0.99%**	**1**
nsp6_161	A	99.01%	100	**V**	**0.99%**	**1**	ORF3a_100	G	99.01%	100	**C**	**0.99%**	**1**
nsp6_181	T	99.01%	100	**I**	**0.99%**	**1**	ORF3a_106	L	99.01%	100	**F**	**0.99%**	**1**
nsp6_195	E	99.01%	100	**E**	**0.99%**	**1**	ORF3a_234	N	99.01%	100	**I**	**0.99%**	**1**
nsp7_11	V	99.01%	100	**I**	**0.99%**	**1**	ORF3d_46	S	99.01%	100	**I**	**0.99%**	**1**
nsp8_76	S	99.01%	100	**F**	**0.99%**	**1**	ORF3d_56	W	99.01%	100	**L**	**0.99%**	**1**
nsp8_180	L	99.01%	100	**F**	**0.99%**	**1**	E_62	V	99.01%	100	**F**	**0.99%**	**1**
nsp9_21	T	99.01%	100	**I**	**0.99%**	**1**	E_68	S	99.01%	100	**F**	**0.99%**	**1**
nsp10_65	Q	99.01%	100	**H**	**0.99%**	**1**	ORF7a_34	P	99.01%	100	**L**	**0.99%**	**1**
nsp10_102	T	99.01%	100	**I**	**0.99%**	**1**	ORF7a_37	S	99.01%	100	**F**	**0.99%**	**1**
RdRp_40	D	99.01%	100	**Y**	**0.99%**	**1**	ORF7a_82	V	99.01%	100	**A**	**0.99%**	**1**
RdRp_179	G	99.01%	100	**C**	**0.99%**	**1**	ORF7a_93	V	99.01%	100	**F**	**0.99%**	**1**
RdRp_250	A	99.01%	100	**P**	**0.99%**	**1**	ORF7a_120	T	99.01%	100	**I**	**0.99%**	**1**
RdRp_613	H	99.01%	100	**Y**	**0.99%**	**1**	ORF8_52	I	99.01%	100	**R**	**0.99%**	**1**
RdRp_678	G	99.01%	100	**C**	**0.99%**	**1**	ORF8_62	V	99.01%	100	**A**	**0.99%**	**1**
RdRp_691	N	99.01%	100	**Y**	**0.99%**	**1**	ORF8_73	C	99.01%	100	**Y**	**0.99%**	**1**
RdRp_930	V	99.01%	100	**A**	**0.99%**	**1**	ORF8_119	D	99.01%	100	**-**	**0.99%**	**1**
Hel_53	P	99.01%	100	**L**	**0.99%**	**1**	ORF8_120	F	99.01%	100	**-**	**0.99%**	**1**
Hel_77	P	99.01%	100	**L**	**0.99%**	**1**	N_3	L	99.01%	100	**D**	**0.99%**	**1**
Hel_260	D	99.01%	100	**N**	**0.99%**	**1**	N_14	R	99.01%	100	**C**	**0.99%**	**1**
nsp14_14	V	99.01%	100	**L**	**0.99%**	**1**	N_35	A	99.01%	100	**T**	**0.99%**	**1**
nsp14_44	G	99.01%	100	**C**	**0.99%**	**1**	N_203	K	99.01%	100	**M**	**0.99%**	**1**
nsp14_119	A	99.01%	100	**V**	**0.99%**	**1**	N_204	R	99.01%	100	**G**	**0.99%**	**1**
nsp14_347	E	99.01%	100	**G**	**0.99%**	**1**	N_208	A	99.01%	100	**V**	**0.99%**	**1**
nsp15_114	T	99.01%	100	**M**	**0.99%**	**1**	N_235	F	99.01%	100	**S**	**0.99%**	**1**
nsp15_205	P	99.01%	100	**S**	**0.99%**	**1**	N_254	A	99.01%	100	**S**	**0.99%**	**1**
nsp15_332	W	99.01%	100	**C**	**0.99%**	**1**	N_383	P	99.01%	100	**S**	**0.99%**	**1**
S_5	L	99.01%	100	**F**	**0.99%**	**1**	ORF9b_75	A	99.01%	100	**V**	**0.99%**	**1**
S_19	T	99.01%	100	**R**	**0.99%**	**1**	ORF14_50	N	99.01%	100	**W**	**0.99%**	**1**

By comparing the 101 Taiwan strains with the WHO's reference Alpha variants (UK-MILK-ACF9CC), we were able to observe 141 amino acid changes during the outbreak. We further calculated the mutation prevalence (in **Boldface text with black shadow**) of these amino acid changes during the outbreak and found that the top prevalence percentages of the two amino acid mutations were 96.04% (97/101) for Helicase-R460K and 86.14% (87/101) for Spike-M1237I presence during the outbreak. Blocks in different colors represent 31 different proteins.

Table S6. Binomial linear regression and Variance Inflation Factors (VIF)

	(1) Epidemic periods	(2) Epicenter	(3) Vaccination coverage	(4) Population size	(5) Population density
1+2+3+4+5	1.553	5.762	1.395	1.891	6.751
**1+2+3+4**	**1.536**	**1.818**	**1.369**	**1.597**	**-**
2+3+4+5	-	1.604	5.895	1.365	5.979

Variance inflation factors (VIF > 5) were used to evaluate collinearity among factors, and the statistically significant factors without collinearity were included in the final multivariable regression model.

Table S7. Multivariable logistic regression (binomial) analysis associated with the frequency of SARS-CoV-2 genome sequences identical to ID-3445/1186/1263 based on Akaike information criterion (AIC) and backward/forward stepwise method to find best-fitting model

	Estimate	Std. Error	z value	Adjusted OR	P value
Identical to ID-3445/1186/1263 = Epidemic periods + Epicenter + Vaccination coverage + Population size, AIC = 86.451					
Epidemic periods	-1.739	0.736	-2.36	0.176	0.018*
Epicenter	-1.477	0.763	-1.94	0.228	0.053.
Vaccination coverage	-0.067	0.691	-0.1	0.935	0.923
Population size	-0.832	0.776	-1.07	0.435	0.284
Identical to ID-3445/1186/1263 = Epidemic periods + Epicenter + Population size, AIC = 84.46					
Epidemic periods	-1.775	0.64	-2.77	0.17	0.006**
Epicenter	-1.499	0.73	-2.06	0.223	0.04*
Population size	-0.82	0.767	-1.07	0.44	0.285
**Identical to ID-3445/1186/1263 = Epidemic periods + Epicenter, AIC = 83.627**					
**Epidemic periods**	-1.738	0.639	-2.72	0.176	**0.007****
**Epicenter**	-1.934	0.606	-3.19	0.145	**0.001****

P value: Fisher's exact test; *: <0.05; **: <0.01.

References

1. Yang, C.R., et al., *FluConvert and IniFlu: a suite of integrated software to identify novel signatures of emerging influenza viruses with increasing risk.* BMC Bioinformatics, 2020. **21**(1): p. 316.
2. Katoh, K. and D.M. Standley, *MAFFT Multiple Sequence Alignment Software Version 7: Improvements in Performance and Usability.* Molecular Biology and Evolution, 2013. **30**(4): p. 772-780.
3. Wu, F., et al., *A new coronavirus associated with human respiratory disease in China.* Nature, 2020. **579**(7798): p. 265-269.
4. Lai, M.M., *Coronavirus: organization, replication and expression of genome.* Annu Rev Microbiol, 1990. **44**: p. 303-33.
5. Kim, D., et al., *The Architecture of SARS-CoV-2 Transcriptome.* Cell, 2020. **181**(4): p. 914-921.e10.
6. Firth, A.E., *A putative new SARS-CoV protein, 3c, encoded in an ORF overlapping ORF3a.* J Gen Virol, 2020. **101**(10): p. 1085-1089.
